# Exploring the role of Yuxuebi tablet in neuropathic pain with the method of similarity research of drug pharmacological effects based on unsupervised machine learning

**DOI:** 10.3389/fphar.2024.1440542

**Published:** 2024-09-17

**Authors:** Xiao Du, Chunhui Zhao, Yujie Xi, Pengfei Lin, Huihui Liu, Shuling Wang, Feifei Guo

**Affiliations:** ^1^ State Key Laboratory for Quality Ensurance and Sustainable Use of Dao-di Herbs, Institute of Chinese Materia Medica, China Academy of Chinese Medical Sciences, Beijing, China; ^2^ College of Pharmacy, School of Medicine, Hangzhou Normal University, Hangzhou, China; ^3^ China Resources Sanjiu Medical and Pharmaceutical Co., Ltd., Shenzhen, China

**Keywords:** traditional Chinese medicine, Yuxuebi tablet, unsupervised clustering, cellular function fingerprint, neuropathic pain

## Abstract

**Introduction:**

Having multiple pharmacological effects is a characteristic of Traditional Chinese Medicine (TCM). Currently, there is a lack of suitable methods to explore and discover modern diseases suitable for TCM treatment using this characteristic. Unsupervised machine learning technology is an efficient strategy to predict the pharmacological activity of drugs. This study takes Yuxuebi Tablet (YXB) as the research object. Using the unsupervised machine learning technology of drug cell functional fingerprint similarity research, the potential pharmacological effects of YXB were discovered and verified.

**Methods:**

LC-MS combined with the *in vitro* intestinal absorption method was used to identify components of YXB that could be absorbed by the intestinal tract of rats. Unsupervised learning hierarchical clustering was used to calculate the degree of similarity of cellular functional fingerprints between these components and 121 marketed Western drugs whose indications are diseases and symptoms that YXB is commonly used to treat. Then, based on the Library of Integrated Network-based Cellular Signatures database, pathway analysis was performed for selected Western drugs with high similarity in cellular functional fingerprints with the components of YXB to discover the potential pharmacological effects of YXB, which were validated by animal experiments.

**Results:**

We identified 40 intestinally absorbed components of YXB. Through predictive studies, we found that they have pharmacological effects very similar to non-steroidal anti-inflammatory drugs (NSAIDs) and corticosteroids. In addition, we found that they have very similar pharmacological effects to anti-neuropathic pain medications (such as gabapentin, duloxetine, and pethidine) and may inhibit the NF-κB signaling pathway and biological processes related to pain perception. Therefore, YXB may have an antinociceptive effect on neuropathic pain. Finally, we demonstrated that YXB significantly reduced neuropathic pain in a rat model of sciatic nerve chronic constriction injury (CCI). Transcriptome analysis further revealed that YXB regulates the expression of multiple genes involved in nerve injury repair, signal transduction, ion channels, and inflammatory response, with key regulatory targets including Sgk1, Sst, Isl1, and Shh.

**Conclusion:**

This study successfully identified and confirmed the previously unknown pharmacological activity of YXB against neuropathic pain through unsupervised learning prediction and experimental verification.

## 1 Introduction

The indications of Chinese patent medicines are mostly described based on traditional Chinese medicine (TCM) theories and terminology, and few of them clearly state the names of modern medical diseases that these medicines can treat (Xie et al., 2020; Wu et al., 2013). However, because TCM has the characteristics of multi-component, multi-target, and multi-pathways, in clinical practice, a Chinese patent medicine is usually used to treat a variety of modern medical diseases. For example, the research object of this article is Yuxuebi Tablet (YXB), a TCM preparation, which is indicated in its instructions to be used for the treatment of Bizheng (arthralgia syndrome) in TCM caused by blood stasis blocking the collaterals. However, YXB has been clinically used to treat many modern diseases and symptoms, including rheumatoid arthritis (RA), osteoarthritis, lumbar disc herniation (LDH), frozen shoulder, cervical spondylosis, etc., despite the lack of clinical and experimental evidence (Chen and Tao, 2018; Fang et al., 2019). Although Chinese patent medicines are widely used to treat various modern medical diseases, they lack the support of clinical and experimental research evidence, and their clinical use is mostly based on the accumulated experience of doctors (Xie et al., 2020; Wang et al., 2019). The experience of doctors in drug application deserves attention, but these experience-based recommendations have an obvious personal drug bias, which is not conducive to the rational use of Chinese patent medicines in clinical practice.

The chemical composition of TCM is complex, and a single compound has the “polypharmacology” properties of multiple targets (Kuenzi et al., 2017). Therefore, TCM has multi-target, multi-pathways, and multi-directional pharmacological effects. Based on this, we put forward a hypothesis: if the Chinese patent medicine X can simultaneously have similar pharmacological effects to multiple types of marketed Western medicines (WMs) for the treatment of modern medical disease Y, X can exert a comprehensive effect through pharmacological effects similar to multiple types of marketed WMs in the treatment of disease Y, and the combination of pharmacological effects may be better than the single effect of WMs. In this way, finding suitable techniques to analyze the similar pharmacological effects of TCM and WMs can become an effective method for discovering modern medical diseases suitable for Chinese patent medicines.

In previous research, we developed an unsupervised hierarchical clustering calculation strategy for TCM based on “biological function similarity evaluation” for the polypharmacology research of TCM (Guo et al., 2021; Guo et al., 2020; Xi et al., 2022). The concept of “cellular function fingerprints” was introduced to indicate the profile of significant therapeutic function influenced by TCM. The similarity of drugs was determined based on the cellular function fingerprints. The unsupervised clustering of drugs based on drug similarity shows that the chemical components of TCM clustered into several modules with WMs, proving that the chemical components of TCM may have similar mechanisms of actions (MoAs) to various WMs, providing a new understanding of the mechanism of TCM in the treatment of complex diseases (Guo et al., 2020). The above research proves that our proposed unsupervised learning strategy is a powerful weapon to solve the problem of comparing the efficacy of TCM and WMs.

**FIGURE 1:**
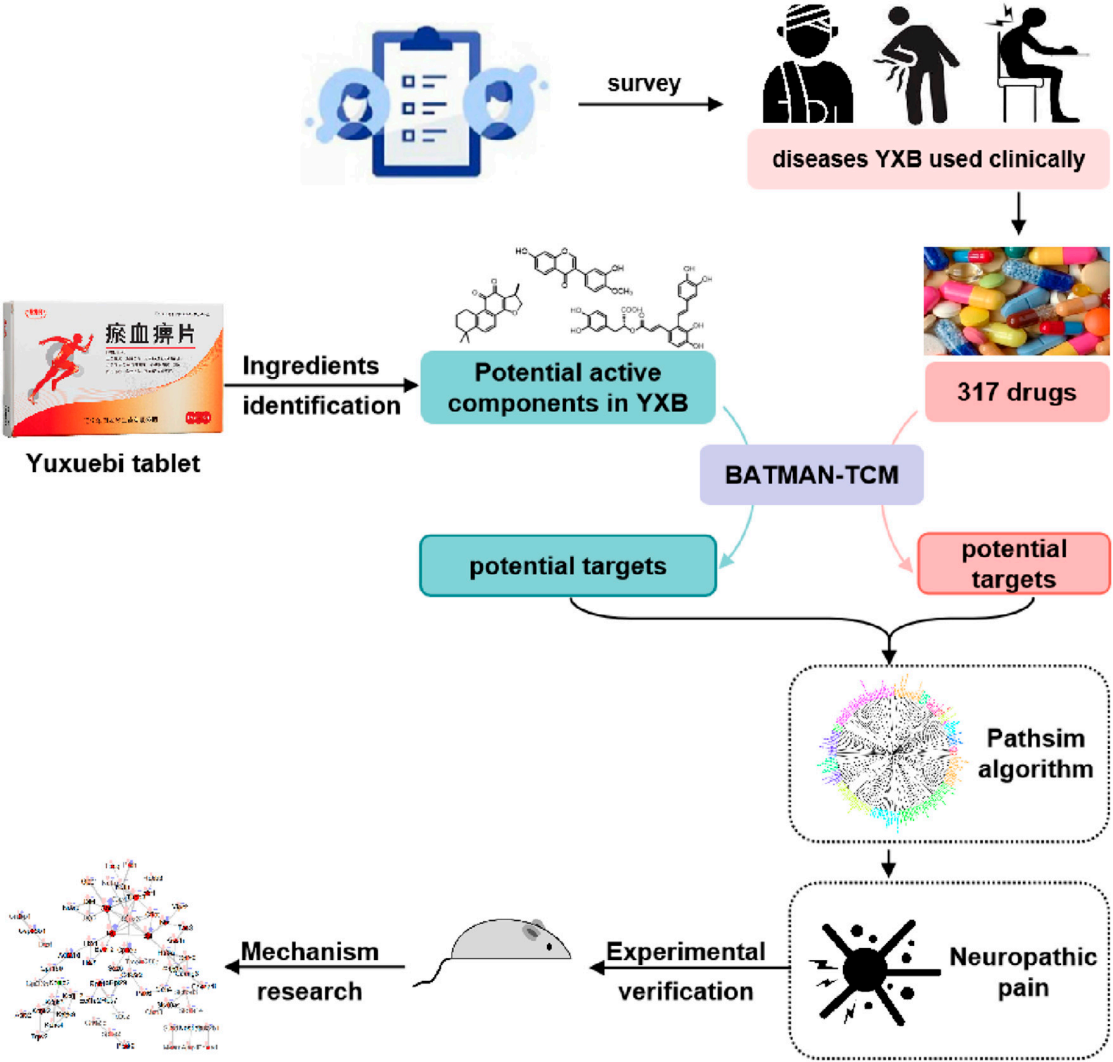
Schematic of the research technology route.

To test the validity of our research ideas, we conducted this research using YXB as an example. YXB is a TCM that has been approved by the Chinese Food and Drug Administration (CFDA). In this study, we included modern medical diseases and symptoms that are recommended by clinicians and for which YXB is clinically suitable for treatment as potential indications for YXB, and collected commonly used marketed WMs for the treatment of these potential indications. Unsupervised machine learning technology was used to investigate the similarity between the chemical components of YXB and the marketed WMs, and to analyze and compare the pharmacological effects of YXB with the WMs. The strategy proposed in this study may help to quickly identify and verify the superior pharmacological effects of TCM and provide research evidence to support its rational clinical application (Figure 1).

## 2 Materials and methods

### 2.1 Identification of the chemical components of YXB

YXB was prepared from the extracts of 11 herbs. The daily amount of medicine taken by the patient was equivalent to the extract prepared from 3.6 g of *Salvia miltiorrhiza Bunge* (*dan shen*), 2.7 g of *Ligusticum chuanxiong* (*chuan xiong*), 2.7 g of *Astragalus* (*huang qi*), 2.7 g of the root of *Clematis chinensis Osbeck* (*wei ling xian*), 2.7 g of *Radix Cyathulae* (*chuan niu xi*), 2.16 g of *Cyperus articulatus* (*xiang fu*), 1.8 g of *Angelica tenuissima* (*dang gui*), 1.8 g Safflower (*hong hua*), 1.8 g of *Curcuma longa* (*jiang huang*), 1.08 g of *Gummi olibanum* (*ru xiang*), and 1.08 g Myrrh (*mo yao*), and the information was provided by the production manager of Liaoning China Resources Benxi Sanyao Co., Ltd., the manufacturer of YXB. In this study, the UHPLC-ESI-MS/MS method was used to identify the chemical constituents of YXB and its absorbed constituents in the isolated intestinal tract of rats. YXB tablets were crushed and ground into powder, dissolved in methanol, and the solution was filtered through 0.22 μm membrane filters before qualitative analysis. Identification of the chemical components in YXB was performed using a Thermo Fisher U3000 ultra-high performance liquid chromatograph (UHPLC) equipped with an on-line degassing device, quaternary gradient pump, column temperature chamber, and automatic sampler, and a Q Exactive PlusTM Orbitrap MS system (Thermo Scientific, Waltham, MA, United States) equipped with a heated electrospray ionization (HESI) source. Chromatographic separation was performed on a Waters ACQUITY UPLC HSS T3 C18 column (2.1 mm × 100 mm, 1.8 μm; Waters Corporation, Milford, MA, United States). UV detection of the UHPLC fractions was performed using a U3000 3D field DAD detector with a wavelength range of 200–400 nm. The analytical column was maintained at 30°C with an injection volume of 5 μL. A gradient elution was performed using a solution of water containing 0.1% (v/v) formic acid in water (solvent B) and acetonitrile (solvent A). The flow rate was 0.2 mL/min, and the gradient elution was as follows: 0–10 min, 100% B; 10–20 min, 100%–70% B; 20–25 min, 70%–60% B; 25–30 min, 60%–50% B; 30–40 min, 50%–30% B; 40–45 min, 30%–0% B; 45–60 min, 0% B; 60–60.1 min, 0%–100% B; 60.1–70 min, 100% B. Positive and negative ion modes in the range of m/z 100–1,500 were set for MS analysis. Other MS operating parameters were a sheath gas flow rate of 40 arb, an auxiliary gas flow rate of 15 arb, a capillary temperature of 320 °C, an auxiliary gas heater temperature of 350°C, a positive spray voltage of 3.2 kv, and a negative spray voltage of 3.0 kv. The MS resolution is 70,000, and the MS/MS resolution is 17,500. Unknown compounds were identified using Compound discover 3.2.0.305 with the mzCloud and mzVault databases.

### 2.2 Identification of the chemical components of YXB that can be absorbed in the rat intestine *in vitro*

The intestinal absorption components of YXB can be considered as potential active components with pharmacodynamic properties in YXB. Therefore, we used an *in vitro* intestinal absorption model to screen the intestinal absorption components of YXB according to previously reported methods (He et al., 2017; Qin et al., 2015). Briefly, rats were fasted for 12 h and euthanized by cervical dislocation. The skin and muscles were cut along the midline of the rat’s abdomen, and the small intestine was quickly removed. The intestine is not less than 10 cm from the pylorus of the stomach and is the jejunum segment. The jejunum (10–24 cm, 34–48 cm, and 58–72 cm from the gastric pylorus) and the ileum (5–19 cm above the ileocecal valve) of approximately 14 cm in length were collected. Each bowel segment was washed with Tyrode’s solution (NaCl 137 mM, MgCl_2_ 1.05 mM, KCl 3.76 mM, NaH_2_PO_4_ 0.42 mM, NaHCO_3_ 11.9 mM, CaCl_2_ 1.8 mM, glucose 5.55 mM; pH 7.4) at 0°C. The silicone tube was inserted into the intestine, and one end of the intestine was tied to the silicone tube with silk sutures, and the intestine was carefully turned over. The inner surface of the intestine was washed with Tyrode’s solution at 0°C, and one end was sutured with silk suture to form a bag.

2 mL of Tyrode’s solution (serosal fluid) was introduced into each inverted pouch, and then the pouches were individually placed in a tube containing 25 mL of oxygenated (O_2_/CO_2_, 95%/5%) Tyrode’s solution with API granules of YXB (0.19 g/mL) on the mucosal side. After incubation for 120 min, the absorption solutions from the four intestinal bags were collected and combined. The absorption solution was then transferred to PE tubes and stored at −80°C until further analysis. We took 0.5 mL of the absorption solutions and used acetonitrile to precipitate proteins at a ratio of absorption solution: acetonitrile of 1:2 and then vortexed them for 1 min, followed by centrifugation at 13,000 rpm for 10 min, and the supernatant was evaporated to dryness on a rotary evaporator. Next, 50 μL of a mixed solution of methanol:water = 80:20 was added. After vortexing for 1 min and sonication for 10 min, the sample was centrifuged at 13,000 rpm for 10 min, the supernatant was collected, and the centrifugation process was repeated once. The supernatant was filtered through 0.22-μm filter membranes and then injected into the UHPLC-MS/MS system for analysis. The unmedicated blank Tyrode’s solution from the four intestinal bags was prepared as a negative control in the same manner as described above.

### 2.3 Prediction of MoA for YXB compounds based on drug similarity evaluation of pathway fingerprints

#### 2.3.1 Drug similarity evaluation based on meta path searching in “compound-target-pathway” heterogeneous network

“Compound-target-pathway” heterogeneous network consists of three kinds of nodes (compound, target, pathway) and two kinds of relationships (compound-target relationship and target-pathway relationship). Compound-target relationship was predicted by BATMAN-TCM (Liu et al., 2016) based on the structure of compounds, whose relationship confidence score is greater than 20. Target-pathway relationship was extracted from gene functional annotation for GO, Reactome and KEGG pathway database. GO annotation database were downloaded in Gene Ontology annotation (GOA, downloaded in August 2021), Reactome pathway database and KEGG pathway annotation database were downloaded in June 2021.

The similarity of pathway fingerprints between two compounds was meticulously assessed in the context of a “compound-target-pathway” heterogeneous network using the PathSim methodology. This method, previously used by recommendation systems in social networks (Sun et al., 2011), was adapted to discover analogous entities that share a common metapath within the complex network (e.g., by identifying compounds with similar pathway profiles). By evaluating the similarity scores of each pair of compounds, a comprehensive similarity matrix for the compounds was formulated. Then, using the R package hClust (hclust function |R Documentation), we performed a hierarchical clustering analysis on the N compounds based on the intricate similarity matrix derived from their pathway fingerprint comparisons. This approach not only highlights the underlying relationships between compounds, but also facilitates the identification of clusters that may share common biological mechanisms or therapeutic potential (Guo et al., 2021).

Regarding the clinically treatable diseases and symptoms of YXB obtained from clinicians, we have conducted a study on the similarity between the active ingredients of YXB and commercial WMs that treat diseases and symptoms from the perspective of drug similarity for each disease or symptom. The distance between the two compounds is 0–1. The smaller the distance, the higher the similarity. WMs whose distances with the potential active ingredients of YXB are less than the median value is included as WMs with higher pharmacological effects similar to YXB, hereafter referred to as similar drugs. The proportion of similar drugs contained in WMs for specific diseases and symptoms was analyzed from three aspects: quantity, pharmacological category, and proportion of clustering groups.

#### 2.3.2 Prediction of ATC code for YXB compounds based on drug similarity evaluation

The most similar WMs of YXB compound can be selected based on similar matrix, whose drug-pathway association can be the possible pathways of YXB compound. YXB compound may have similar ATC code of the selected WM.

#### 2.3.3 Prediction of targeted GO terms for YXB compounds based on drug similarity evaluation

YXB compound may have similar GO terms of the most similar WM. By extracting the meta-path between YXB compound and the most similar WM from the “drug-target-pathway” network, targeted GO terms for YXB compounds were predicted from the common GO annotation term between YXB compound and the most similar WM. Targeted GO terms for WMs were calculated from the sharing GO annotation term between WMs.

### 2.4 Prediction of YXB’s indication based on MoA coverage of YXB to WMs

To evaluate the pharmacological effect similarity between YXB and WMs, the pharmacological effect coverage based on ATC code, GO terms and drugs were calculated. And a combined coverage score called CombinedRatio as an auxiliary analysis index. The CombinedRatio is calculated by combining the probabilities from the three types of evidence sources as follows:

$$\text{CombinedRatio}=\frac{\text{ATC}\;\text{code}\;\text{coverage}+\text{GO}\;\text{term}\;\text{coverage}+D\text{rug}\;\text{coverage}}{3}$$

#### 2.4.1 WMs for diseases and symptoms and MoA effect

We conduct a questionnaire to investigate the frequent indications of YXB for 64 clinicians from 16 hospitals in Beijing and Shanghai, China. The detailed information of the hospitals and the distribution of these clinicians’ departments are listed in Supplementary Table S1 and Supplementary Table S2. WMs for these indications were searched in the global drug research and development database of Yaozh.com (https://db.yaozh.com/?=yaozh), clinical disease treatment guidelines and literature. The ATC code of these drugs was also collected from Yaozh.com to indicate the pharmacological effect.

The InChI strings of the collected WMs were retrieved from the PubChem database. For those compounds not found in PubChem, we use ChemDraw software to generate their structure, we also use OpenBabel to get the InChI strings.

#### 2.4.2 YXB’s pharmacological effect coverage for specific disease or symptom based on ATC code

For each indication, ATC code for YXB compounds were predicted based on drug similarity evaluation. The set of ATC code of YXB were used to indicate the potential pharmacological effect of YXB. The set of ATC code of WMs in this indication was collected, which was described in 2.4.1. If the WMs for this indication belong to N ATC codes, YXB was predicted to relate to n ATC codes. The ***ATC code coverage*** is the ratio of the number of predicted ATC codes of YXB for the treatment of disease or symptom X.

$$\text{ATC code coverage}=\frac{n}{N}$$

For the complex disease, WMs were developed for different MoAs of the indication. As a result, the ATC code set of WMs represents the MoAs of that indication. The ATC code coverage of YXB implies YXB coverage for the complex MoAs of this indication.

#### 2.4.3 YXB’s pharmacological effect coverage for specific disease or symptom based on target GO terms

For each indication, target GO terms for YXB compounds were predicted based on drug similarity evaluation and meta-path searching. The set of GO terms of YXB was used to indicate the potential pharmacological effect of YXB. The set of GO terms of WMs in this indication were collected, which was described in 2.3.3. If the target of WMs for this indication have *M* GO terms, YXB were predicted to relate to *m* GO terms, ***GO term coverage*** represents the ratio of the number of predicted targeted GO terms of YXB for the treatment of disease or symptom X.

$$\text{GO term coverage}=\frac{m}{M}$$

YXB′s ***GO term coverage*** is another measure of the coverage of the complex MoAs in this indication.

#### 2.4.4 YXB’s pharmacological effect coverage for specific disease or symptom based on similar WMs

For an indication, there are D WMs used for treatment. Among them, *d* WMs were clustered to YXB compounds after similarity evaluation. ***Drug coverage*** represents the ratio of the number of WMs similar to the active ingredients of YXB for treating disease or symptom X.

$$D\text{rug coverage}=\frac{d}{D}$$

The ***Drug coverage*** of YXB is another index to show the coverage of the complex MoAs of this indication.

### 2.5 Potential pharmacological mechanism study of YXB based on drug similarityPotential pharmacological mechanism study of YXB based on drug similarity

RA, for which it has been clinically confirmed that YXB has a good therapeutic effect (Zhang et al., 2020), and LDH, which ranks high in the above similarity studies and appears more frequently in the YXB clinician survey, were selected for further research. In addition, because our team is conducting a clinical trial of YXB for the treatment of nocturnal pain in ankylosing spondylitis (AS) (Trial Registration: Unique Protocol ID 2020005P8A02 ClinicalTrials.gov ID NCT04934059), we included AS in this part of the study. We adopted the research method described in Section 2.4 to analyze the drug similarity between the YXB and WMs components for the treatment of the two diseases. We analyzed the pharmacodynamic mechanism of YXB with the transcriptomic data obtained from the Library of Integrated Network-based Cellular Signatures (LINCS) (Keenan et al., 2018), and used the MoA of similar drugs as clues to identify the MoA of YXB in the treatment of diseases and symptoms. During the research, we first performed another cluster analysis of the similar drugs and YXB components of the selected disease or symptom using the Pathsim algorithm. Based on the “compound-target-biological function” network, the biological processes in which both similar drugs and YXB components can intervene are extracted, which is called common biological processes (Guo et al., 2020). Then, we obtained the transcriptome data of similar drug intervention cells from LINCS and analyzed the regulatory direction of drugs on common biological processes.

### 2.6 Analgesic effect and mechanism of YXB in rats with CCI

#### 2.6.1 Animals and treatment

Adult male Wistar rats (8 weeks old, body weight 250–300 g) were randomly divided into (1) sham group; (2) CCI group; (3) CCI + YXB-0.31 g/kg; (4) CCI + YXB-0.62 g/kg; (5) CCI + YXB-1.25 g/kg. Each group contained 10–13 rats. The raw material powder of YXB was provided by the drug manufacturer (China Resources Benxi Third Pharmaceutical), and 1-g tablet contained approximately 0.936 g of raw material powder. The animals were allowed to eat freely during the experiment, and all experimental procedures strictly followed the relevant regulations of animal welfare and ethics. After surgery, the success of the CCI model was confirmed by the behavioral experiment. Starting on postoperative day 5, different drugs were administered by gavage once a day until postoperative day 16.

#### 2.6.2 Experimental design

The CCI rat model was prepared according to the method described in the literature (Bennett and Xie, 1988). After the animals were anesthetized, the sciatic nerve trunk of the left leg of each rat was surgically isolated and exposed, and four chromic catgut sutures were evenly ligated to the sciatic nerve trunk at 1 mm intervals. The ligature was applied with a force that caused a slight vibration of the rat’s thigh muscles or a slight vibration of the left foot. The skin was sutured and sterilized. The operation of the animals in the sham-operated group was the same as that of the CCI group, except that the sciatic nerve was not ligated. On the day before surgery and on the 3rd, 5th, 7th, 10th, 13th, and 16th days after surgery, the mechanical withdrawal threshold (MWT) of the rats in each group was evaluated by the von Frey test, and the thermal withdrawal threshold (TWT) of the hind paw of the rats was evaluated by irradiation with a thermal radiation stimulator. In addition, we assessed persistent nociception in rats by observing limb use during spontaneous walking. The use of the affected limb during the animal’s voluntary activities was scored on a scale of 0–4: 0, complete inability to use the limb; 1, relative inability to use the limb; 2, marked lameness and defensive behavior; 3, mild lameness; 4, the toes on the affected side are closed when walking; and 5, normal use (Bao et al., 2015).

#### 2.6.3 RNA-seq analysis

Two weeks after surgery, all animals were decapitated. Spinal cord total RNA was extracted with TRIzol reagent (TIANGEN, China) and detected on a 1% agarose gel. Purity, concentration, and integrity of the total RNA samples were evaluated before further analysis. After clustering, the library preparations were sequenced on the Illumina HiSeqTM 4,000 platform from Biomarker Technologies (Beijing, China), and raw reads were generated. Raw reads were filtered by removing adapter and poly-N sequences and low-quality reads. The clean reads were mapped to the *Rattus norvegicus* reference genome sequence using the HISAT2 software. Quantitative gene expression levels were estimated by determining the number of fragments per kilobase of transcripts per million mapped fragments. Gene expression analysis of the different groups was performed using DESeq2. Genes with a P value < 0.1 were defined as differentially expressed genes (DEGs). Enrichment analysis of the DEGs was then performed using the KOBAS3.0 platform. Volcano plots and heat maps were generated using OmicShare online tools (http://www.omicshare.com/tools). Venn diagrams were generated using the Draw Venn Diagrams online tools.

## 3 Results

### 3.1 Identification of potential active compounds of YXB

**FIGURE 2:**
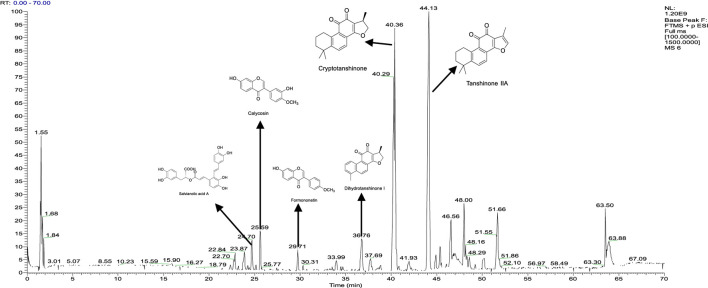
Base Peak Chromatogram in the positive mode of processed-YXB.

The chemical compounds of YXB were identified by HPLC-MS/MS. The liquid phase diagram is shown in Supplementary Figure S1, and the ion flow result is shown in Figure 2, which includes the identification results of some compounds. 344 compounds were identified or tentatively identified by HPLC-MS/MS based on retention time, MS/MS spectra and fragmentation behavior. The HPLC-MS/MS information (such as retention time, chemical formula, and ppm error) are summarized in Supplementary Table S3. To detect the active ingredients that can be absorbed in YXB, we collected the components that can be absorbed in the isolated rat small intestine from YXB and identified them by HPLC-MS/MS. The result of ion flow is shown in Supplementary Figure S2. We identified 116 chemical components of YXB from the prepared intestinal juice, which are considered to be potential active components with pharmacodynamic properties of YXB, and the results are shown in Supplementary Table S4. It can be seen that the components of YXB and the components that can be absorbed in the intestine contain many types of compounds, including phenanthrenequinones, organic acids, alkaloids, flavonoids, esters, phthalides, curcumin, glycosides, sugars, etc., among which salvianolic acid and tanshinone compounds are the main components.

### 3.2 Similarity analysis of potential active ingredients of YXB and the commercial WMs for various diseases and symptoms based on unsupervised hierarchical clustering

To understand whether YXB can be used to treat certain modern medical diseases and symptoms, we visited physicians in various departments of a number of hospitals and learned that YXB is used by physicians to treat more than 20 modern medical diseases and symptoms, including osteoarthritis, LDH, post-fracture swelling and pain, and cervical spondylosis, as well as rheumatic diseases such as gout, RA, and AS (all diseases and symptoms are shown in Supplementary Table S5). We collected commonly used WMs for the treatment of each disease and symptom. In the end, we collected a total of 317 drugs approved for the treatment of clinical indications of YXB, the data are shown in Supplementary Table S6.

Hierarchical clustering was used to reveal clusters of compounds with similar attributes. Similarity between two compounds was determined from cellular function fingerprints using the hclust function in R (Guo et al., 2020). We obtained all potential targets of the compound using the prediction resource BATMAN-TCM (Liu et al., 2016), and targets with scores greater than 10 were considered potential targets of the query compound. Due to the large number of WMs collected, to improve the accuracy of the results, before comparing the similarity of the active ingredients of WMs and TCM, we first performed a consistency analysis of all the WMs collected. The results of the cluster analysis of the similarity of the cellular function fingerprints based on the GO Biological Processes, Reactome, and Wiki Pathways of these 317 drugs are shown in Supplementary Figure S3. The pharmacological effects of drugs with the same MoA should be closely clustered. Therefore, we analyzed the similarity of the cellular function fingerprints between WMs and eliminated drugs that were not under the same cluster tree branch with other drugs (i.e., the similarity is low). After screening, 121 WM types were retained and divided into 28 categories based on their ATC classification and pharmacological effects (Supplementary Table S7).

**FIGURE 3:**
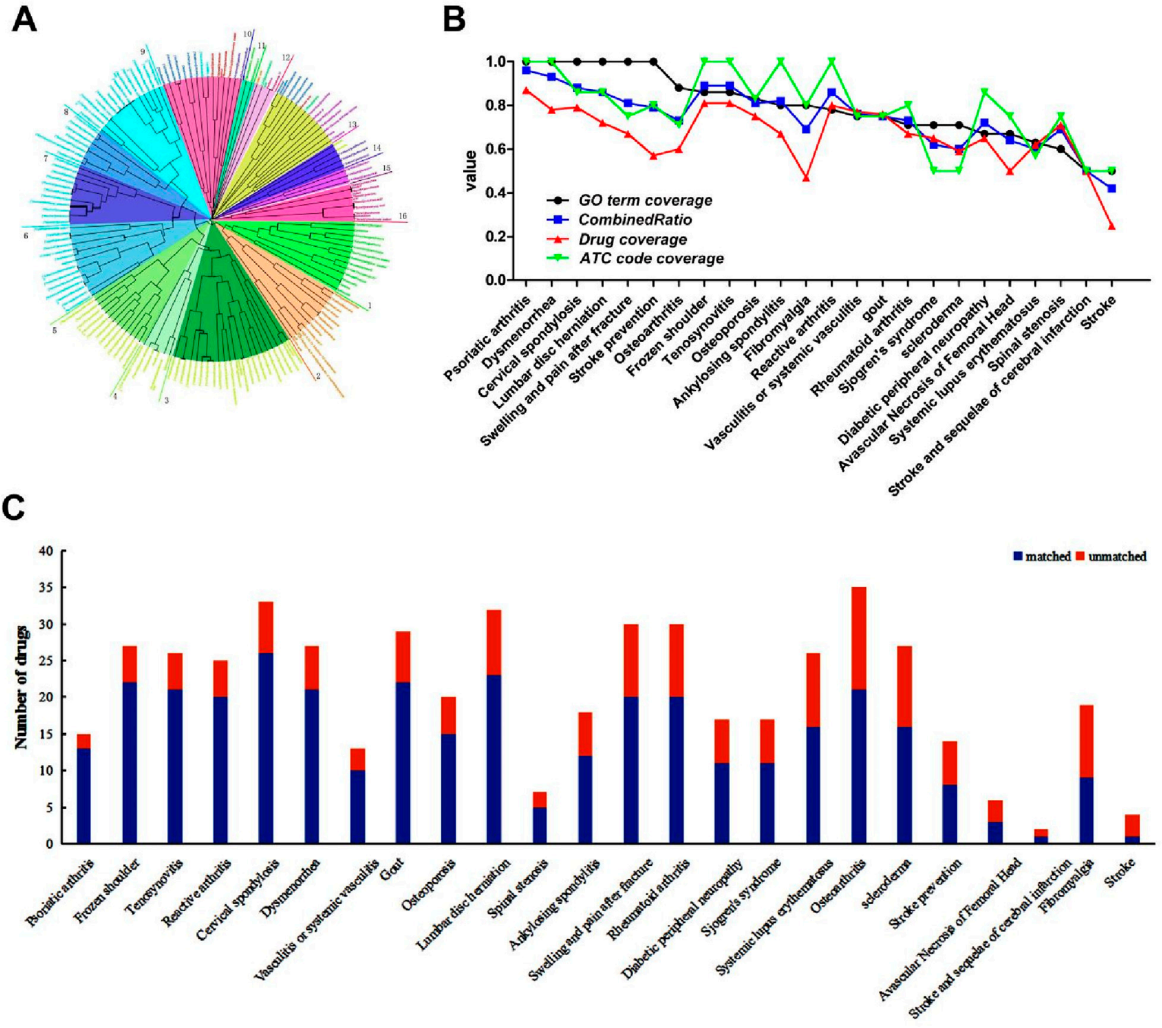
Results of drug similarity between the components of YXB and WMs for the potential indications of YXB. **(A)** Unsupervised hierarchical clustering of YXB compounds and commercial WMs for different diseases and symptoms; **(B)** Similarity ratio results of YXB and WMs for different diseases and symptoms; **(C)** Statistics on the number of similar drugs contained in WMs for different diseases and symptoms. Matched refers to the number of similar drugs; unmatched refers to the number of non-similar drugs.

A total of 40 potential active compounds of YXB were included after screening by the target scoring value, and similarity studies were performed on these compounds with the 121 WMs screened above. Hierarchical clustering of compounds was used to display similarity distances and groups of all compounds. A “compound-target-cellular function” heterogeneous network was used to evaluate the similarity of functional fingerprints between compounds using the Pathsim algorithm. The results are shown in Figure 3A. Hierarchical clustering of TCM compounds based on similarity can help to classify compounds into multiple modules with similar therapeutic functions to study the multicomponent synergistic mechanisms of TCM. The cluster diagram showed that the clustering tree branches of all the compounds can be divided into 16 groups, among which groups 1, 2, 4, 6, 9–11, 13, and 14 contain both TCM and WM ingredients, among which 90% of the YXB compounds could aggregate with WMs. The potential active ingredients of YXB could be clustered under different clustering tree branches with different WMs, indicating that the components of YXB have similar pharmacological effects with different WMs.

The median value of the similarity distance between the potential active ingredients of YXB and the WMs was 0.53, and highly similar WMs with similarity distances of less than 0.53 were screened. The results showed that the components of YXB had high similarity with several drugs in the WMs for each disease and symptom, and these similar drugs were distributed in different clustering groups, indicating that YXB may have different pharmacological effects on different diseases and symptoms. The results of ***GO term coverage***, ***Drug coverage***, ***ATC code coverage***, and CombineRatio are shown in Figure 3B, and detailed data are provided in Supplementary Table S8. It can be seen that the WMs for the treatment of each disease and symptom were clustered in different regions of the clustering tree according to the different cell function fingerprints and were divided into different clustering groups. Taking LDH as an example, a total of 32 WMs for the treatment of LDH were included in the analysis library, and were divided into 7 cluster groups according to their distribution in the clustering tree. WMs with a similarity distance of less than 0.53 with the potential active components of YXB were screened, and 23 of the 32 WMs for the treatment of LDH were similar drugs, as shown in Figure 3C, which were distributed in 7 clustering groups; that is, the ***Drug coverage*** was 0.72 for 23/32, and the ***GO term coverage*** was 1 for 7/7. It can be predicted that the similarity between the pharmacological effects of YXB components and those of WMs for the treatment of a particular disease and symptom is to some extent positively related to the ***GO term coverage***. The top ten indications with the highest ***GO term coverage*** are: psoriatic arthritis, dysmenorrhea, cervical spondylosis, LDH, swelling and pain after fracture, stroke prevention, osteoarthritis, frozen shoulder, tenosynovitis, and osteoporosis.

### 3.3 Possible pharmacological mechanism of YXB in the treatment of LDH, RA and AS

**FIGURE 4:**
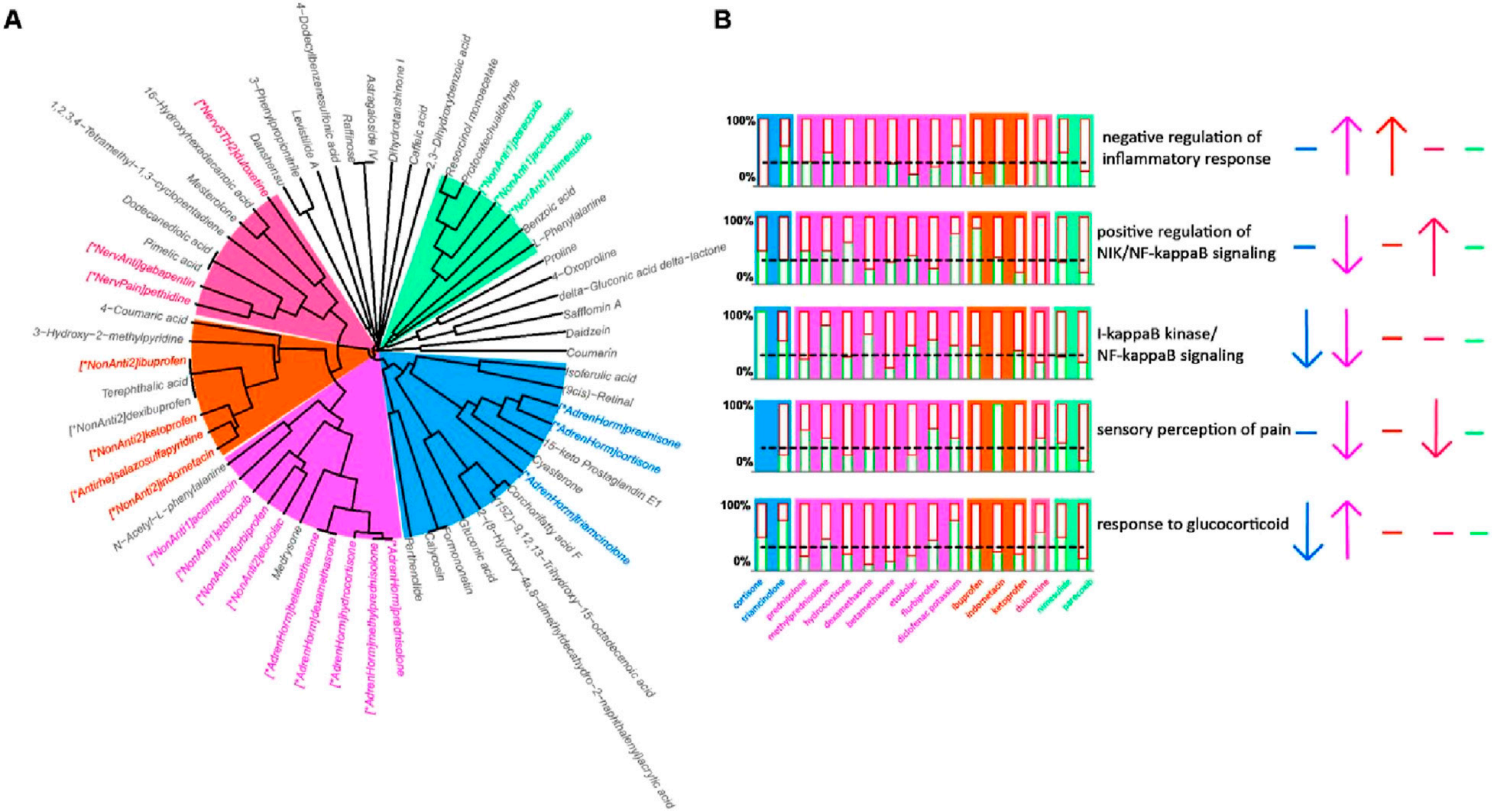
Potential biological process regulation mechanism of YXB in the treatment of LDH, RA and AS. **(A)** Unsupervised hierarchical clustering of YXB compounds and drugs for the treatment of LDH, RA and AS; **(B)** Common pathways and the regulatory effects of WMs on them. Based on the clustering results, different drug groups were marked with different colors. In the column diagram of **(B)**, if the column marked with a red border down crosses the gray dashed line, it means that the drug has a positive regulatory effect on this biological process. On the contrary, if it does not cross the gray dashed line, it means that the regulatory effect is negative. On the right side of **(B)**, the up arrow means that this group of drugs has an up-regulatory effect on this biological process from the overall assessment, the down arrow means a down-regulatory effect, and the horizontal line means that the overall regulatory effect is flat.

To investigate the pharmacological mechanism of YXB, we selected LDH, AS, and RA (***GO term coverage*** was 1, 0.80, and 0.71, respectively) for further research, and investigated the biological processes that can be regulated by both WMs and YXB components (hereafter referred to as common pathways). The 121 WMs screened out in the previous step included 22 WMs for LDH, 13 WMs for RA and 12 WMs for AS (see Supplementary Tables S6 and S7 for details). We used the Pathsim algorithm to investigate the drug similarity between the potential active components of YXB and the WMs for the treatment of LDH, RA and AS. The results are shown in Figure 4. The drugs in the colored font in Figure 4A are similar drugs that can be clustered under the same cluster tree branch with YXB components. They are divided into five groups from 1 to 5 according to the clustering situation, and are marked with different colors. It can be seen that the WMs included in the first group are all adrenocorticoid steroids, the fourth group are all drugs that act on the nervous system, the fifth group are all non-steroidal anti-inflammatory drugs (NSAIDs), the third group contains 3 NSAIDs and one anti-rheumatic drug, and half of the drugs included in group 2 are adrenocorticoid steroids and half are NSAIDs. The common pathways are shown in Figure 4B. And the regulation direction of regulation of WMs on common pathways was investigated based on transcriptome data from LINCS. As shown in Figure 4B, the regulatory effects of similar drugs on common pathways included promotion of negative regulation of inflammatory response, inhibition of I-κB kinase/NF-κB signaling and pain sensation. And only adrenocortical hormone drugs could activate the response to glucocorticoid, indicating that LINCS transcriptome data can exhibit the MoA characteristics of WMs. Combined with the pharmacological effects of similar drugs, it is speculated that YXB components should be able to inhibit inflammation, NF-κB signaling pathway, and pain sensation in the treatment of LDH, RA, and AS.

From the clustering results, we extracted the WMs most similar to each compound of YXB, and the pairs of compounds with more than 0.5 similarity are listed in Table 1. From the results, it can be seen that the types of WMs with the highest similarity to YXB are mainly anti-inflammatory, immunosuppressive and anti-rheumatic drugs. For example, *dang gui*-derived levistilide A and *dan shen*-derived protocatechualdehyde in the prescription of YXB showed high biological function similarity with the non-steroidal anti-inflammatory drugs mizoribine and parecoxib, respectively. Literature reports proved that levistilide A and protocatechualdehyde have immunosuppressive activity, which also proves the accuracy of our research (Xiong et al., 2018; Yang et al., 2021).

**TABLE 1:** List of the WMs with the highest degree of similarity to the potential active ingredients of YXB.

Potential active compound of YXB	Most similar WM	Similarity	Pharmacological effect
Terephthalic acid	nicotinic acid	1	Decrease blood lipids (Hall et al., 1993)
4-coumaric acid	Imidazole salicylate	1	Immunomodulatory, anti-inflammatory, neuroprotective effect, etc. (Zhu et al., 2018; Abdel-Moneim et al., 2017; Kheiry et al., 2020)
N-Acetyl-L-phenylalanine	actarit	0.97	
[dang gui] Dodecanedioic acid	sodium valproate	0.96	Alternative energy substrate (Mingrone et al., 2013)
Pimelic acid	sodium valproate	0.96	Energy substrate (Mingrone et al., 1989)
15-keto Prostaglandin E1	alprostadil	0.91	Reduce thrombosis (Sinzinger et al., 1998)
[dang gui] Levistilide A	rofecoxib	0.86	Antithrombotic, anti-inflammatory, anti-liver fibrosis, etc. (Zhang et al., 2021; Li et al., 2020a)
[dan shen/huang qi] Isoferulic acid	febuxostat	0.83	Anti-oxidation, anti-glycation (Meeprom et al., 2015; Meeprom et al., 2018)
[huang qi] 3-Hydroxy-2-methylpyridine	glucosamine	0.82	Inhibit fibril formation (Mariño et al., 2015)
Resorcinol monoacetate	parecoxib	0.82	Cholinergic action (Moritoki and Ishida, 1977)
[hong hua] Safflomin A	mizoribine	0.79	Suppress immunity (Lu et al., 1991)
[dan shen] Protocatechualdehyde	parecoxib	0.76	Anti-oxidation, anti-inflammatory, neuroprotective, etc. (Yang et al., 2021; Guo et al., 2019)
[chuan niu xi] Cyasterone	cortisone	0.73	Accelerates fracture healing (Zhu et al., 2021)
Medrysone	hydrocortisone	0.71	Anti-inflammatory (Minigh, 2007)
[ru xiang] (9cis)-Retinal	ergocalciferol	0.63	
3-Phenylpropionitrile	cilostazol	0.54	
Mesterolone	sodium valproate	0.52	Raise HDL (Casquero et al., 2017)

### 3.4 YXB attenuated paw mechanical and thermal hypersensitivity and gait abnormalities after CCI surgery

**FIGURE 5:**
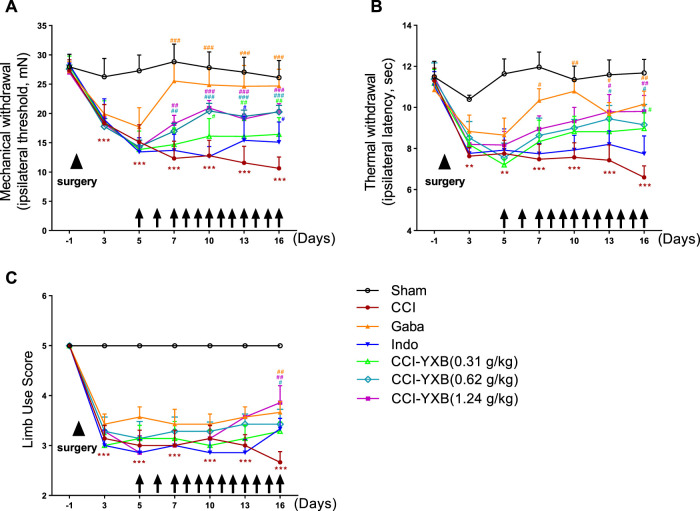
YXB reduced mechanical allodynia, thermal hyperalgesia, and gait abnormalities induced by CCI surgery. Starting on postoperative day 3, rats in the CCI group showed a significant reduction in mechanical **(A)** and thermal **(B)** withdrawal thresholds and limb use scores **(C)** that lasted for at least 16 days. Different drugs were administered on postoperative day 5, and various behavioral indices were improved to different degrees.

Compared with the sham group, the mechanical pain threshold (*p* < 0.001), thermal pain threshold (*p* < 0.01) and gait score (*p* < 0.001) of the animals in the other groups were significantly decreased on the third day after surgery, and there was no significant difference between the groups, indicating that the animal model was successfully established, and the grouping is uniform, and subsequent experiments can be conducted and the result is shown in Figure 5. It can be observed that the mechanical pain threshold and thermal pain threshold of the rats in the CCI group showed a continuous downward trend throughout the experimental period. Each dose group of YXB significantly improved various pain behavior indices in CCI rats, among which the improvement effect on mechanical pain threshold was the most significant, and the drug efficacy showed a certain dose-effect and time-effect correlation. From the 7th day after surgery, the MWT of the animals in the middle and high dose administration groups of YXB administration increased significantly compared with the model group (*p* < 0.01), and the MWT of the animals in the low dose group increased significantly from the 10th day after surgery (*p* < 0.05). After administration, the TWT of animals in each dose group of YXB showed a continuous upward trend, and significantly increased significantly to varying degrees on the 13th or 16th day (*p* < 0.05). The improvement effect of the therapeutic drugs on the limb use of the animals was relatively slow, and the administration of gabapentin and medium and high doses of YXB did not significantly improve the limb use scores until the 16th day after surgery. The MWT and TWT of the animals in the gabapentin group increased significantly from the 7th day after surgery compared with the model group (*p* < 0.05). Only on postoperative days 9 and 12 a significant increase in MWT was observed after indomethacin administration (*p* < 0.05).

### 3.5 Identification and functional analysis of differentially expressed genes

**FIGURE 6:**
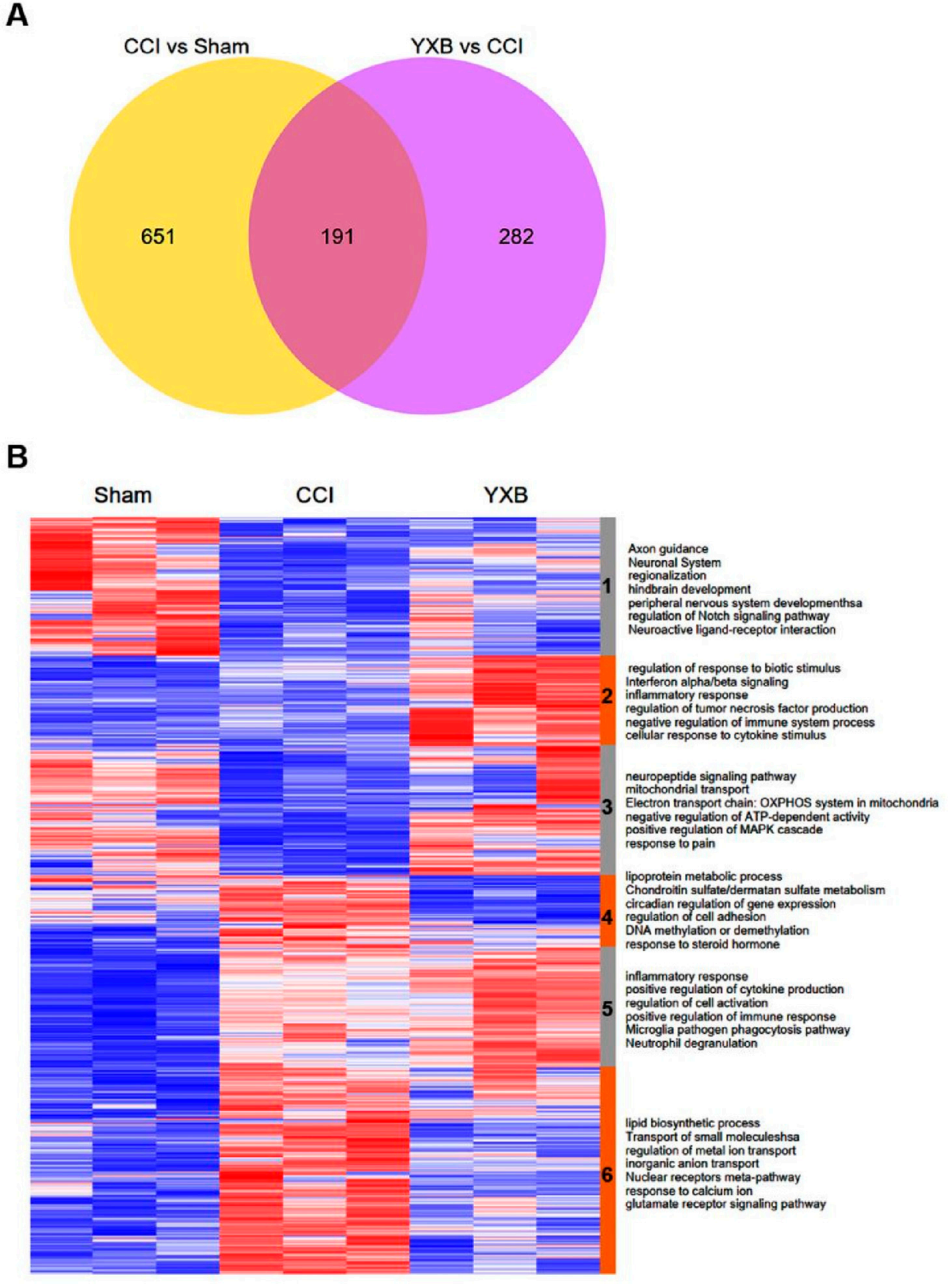
mRNA expression profiles in spinal cords of different groups of animals. **(A)** Venn diagram of differentially expressed mRNA between different groups. **(B)** Up- and downregulated genes are shown in red and blue, respectively. According to the different expression patterns, all genes were divided into 6 groups from top to bottom, and the high GO enrichment terms of genes in each group were marked on the right. n = 3 animals per group.

Transcriptome detection of animal spinal cord showed that there were a total of 842 differentially expressed mRNAs between CCI group and Sham group. There were 473 differentially expressed mRNAs in the YXB group compared to the CCI group. In total, there were 191 overlapping differentially expressed mRNAs, as shown in Figure 6A. Hierarchical heat map analysis of differentially expressed mRNAs revealed clustering patterns of the differential mRNA expression in different groups, as shown in Figure 6B. According to the different gene expression patterns, the genes can be divided into 1–6, 6 groups, as shown in Figure 6B. Group 1, 3, 4, and 6 were the groups in which the trend of gene expression in the CCI group was significantly reversed after YXB administration. GO functional enrichment analysis showed that the genes in group 1 were mainly related to GO terms in neural development, the genes in group 3 were mainly related to mitochondrial energy metabolism, the genes in group 4 were mainly related to cell adhesion, and the genes in group 6 were mainly related to ion channel.

**FIGURE 7:**
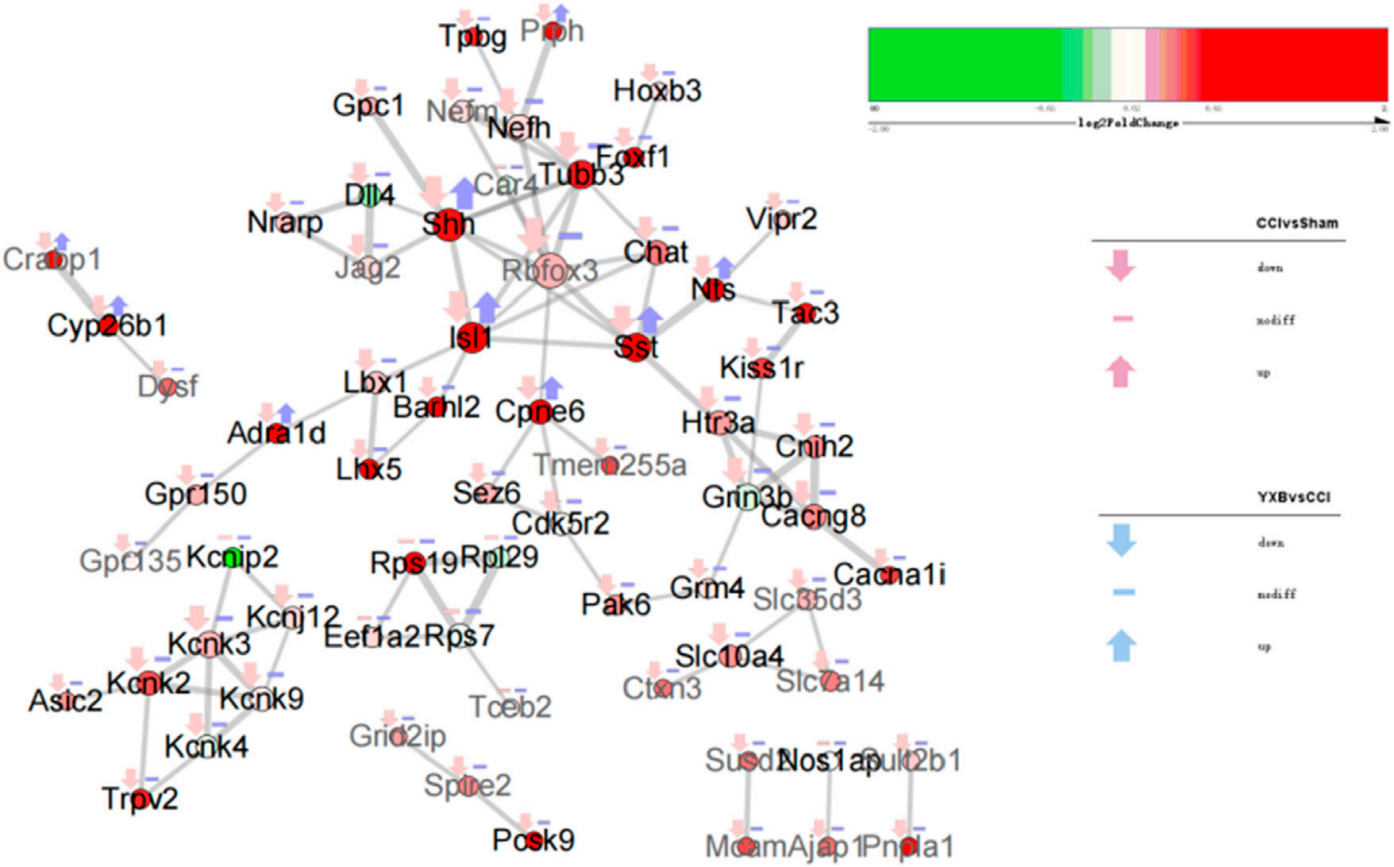
The protein interaction network diagram of cluster heat map group 1 genes. Each node in the figure represents a protein, and each connecting line represents the interaction between the connected proteins. The size of the node is positively correlated with the degree of connectivity. The darker the fill color, the higher the expression difference of the gene corresponding to the node protein in the YXB group vs. CCI group. The genes corresponding to black fonts are shown to be related to neurodevelopment in the GO database, and the genes corresponding to gray fonts are not found to be related to neurodevelopment in the GO database. Differential gene annotation: up arrows indicate significantly upregulated genes that satisfy FoldChange > 1.5 and padj < 0.1; down arrows indicate significantly downregulated genes that satisfy FoldChange > 1.5 and padj < 0.1; dashes indicate genes that do not satisfy the screening conditions; pink indicates CCI vs. Sham group, blue is YXB group vs. CCI group.

**FIGURE 8:**
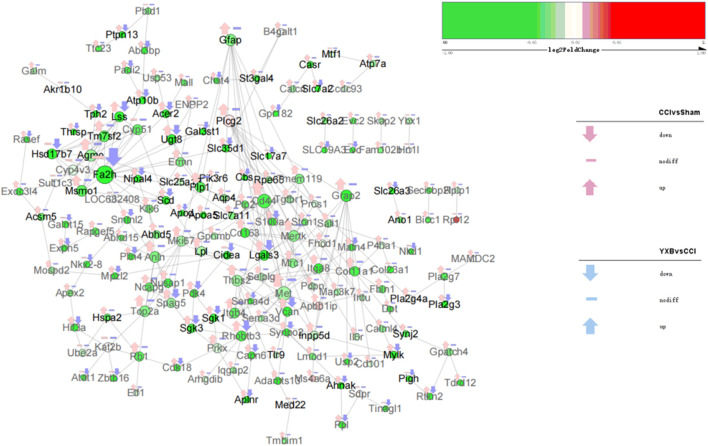
The protein interaction network diagram of cluster heat map group 6 genes. The genes corresponding to black fonts are shown to be related to ion channels in the GO database, and the genes corresponding to gray fonts are not found to be related to ion channels in the GO database. Other legends have the same meaning as in Figure 7.

As shown in Figure 6B, the gene expression levels in group 1 and group 6 were significantly reversed after YXB intervention compared with the CCI group, and GO enrichment analysis showed that these two groups of genes were closely related to neuropathic pain. To explore the mechanism of YXB against neuropathic pain, we constructed and visualized the protein-protein interaction (PPI) network of genes in group 1 and group 6 (Figures 7, 8). According to the degree of connectivity of the network and the significance of differential expression of genes, Isl1, Sonic hedgehog (Shh), Sst, Cpne6, Nts and Tubb3 were identified as the major hub genes of the PPI network of genes in group 1. In group 6, serum and glucocorticoid-inducible kinase 1 (Sgk1), Lss, Lgals3, Ugt8, Sgk3, Acer2 and Fa2h were identified as major hub genes, and YXB intervention was able to suppress the expression of these genes. These major hub genes were significantly up- or downregulated in the CCI group, but YXB intervention could significantly reverse the trend of their expression levels. In addition, although the expression of Fa2h in group 6 was not significantly upregulated in the CCI group, but it was significantly downregulated in the YXB group and had the highest degree of connectivity in the network, which deserves further attention. GO database search revealed that major hub genes in group 1 were related to neurodevelopmental processes, while major hub genes and other genes of interest in group 6 were related to ion channel processes.

## 4 Discussion

The description of the indications of Chinese proprietary medicines lacks specific modern medical disease terminology, which leads to the failure to rationally guide Western medical practitioners to use Chinese medicines, which severely hinders Chinese medicines from exerting their clinical value (Hao et al., 2017; Mao et al., 2007). To explore an objective and effective method for evaluating the modern disease indications of Chinese patent medicines. This study proposes a hypothesis that due to the complex pharmacological action pathways of TCM, if a Chinese patent medicine can produce similar pharmacological effects to various marketed WMs in the treatment of a specific disease, it can potentially treat the disease through a similar pharmacological action as these WMs. In this study, referring to the previous study (Guo et al., 2020; Xi et al., 2022), we used the artificial intelligence unsupervised machine learning cluster analysis method to study the similarity between the potential active components of YXB and the pharmacological effects of many different WMs, and analyzed the pharmacological effects of YXB, which is a prescription Chinese medicine that has been on the market in China for many years and its indications are expressed in terms of TCM.

The clinical medication guidelines and textbooks recommend the use of YXB for the treatment of RA, scleroderma, osteoarthritis and other modern medical diseases and symptoms based on the theory and medication experience of Chinese medicine, but lack sufficient support from efficacy evaluation data (Chen and Tao, 2018; Fang et al., 2019; Yan et al., 2015; Dong et al., 2018). Clinicians have rich clinical experience in the use of YXB, which provided a number of valuable candidate diseases and symptoms for the discovery of suitable modern medical indications of YXB. After visiting doctors, we learned that YXB may be able to treat more than 20 modern medical diseases and symptoms including RA, scleroderma, osteoarthritis, etc. However, this study lacked a more rigorous design for collecting potential indications for YXB during the implementation process, and a more reliable experience of drug use would have been obtained by adding more dimensional and quantifiable indicators, such as: gender, age and title of the doctor, grade and size of the hospital where he/she works, frequency of use of YXB, years of use of YXB, etc., as well as quantitatively designing the questions of the interview questionnaires. Then, we tried to analyze the rationality of using YXB in clinical treatment of various diseases and symptoms from the perspective of the commercial WMs with higher similarity to the active ingredients of YXB. Using unsupervised machine learning cluster analysis technology, we compared the similarity of the pharmacological effects of the potential active ingredients of YXB and the WMs for the disease and symptom treated by YXB (Guo et al., 2021).

The similarity of pharmacological effects between drugs showed that YXB has most of the pharmacological activities of WMs for these diseases and symptoms, and exerts effective therapeutic effects through comprehensive effects. Diseases and symptoms with high ***GO term coverage*** are mostly inflammatory and painful diseases and symptoms, such as psoriatic arthritis, dysmenorrhea, cervical spondylosis, etc. Studies have shown that YXB has shown good anti-inflammatory and analgesic effects in animal experiments, and can increase the pain threshold and reduce foot swelling in mice with chronic inflammatory pain by inhibiting the expression of peripheral inflammatory factors such as TNF-α, IL-17A and CCL2 (Li R. et al., 2020; LI et al., 2021). The treatment of diseases by TCM is characterized by the synergistic effect of multiple components. We performed a hierarchical clustering analysis of compounds by conducting pathway-based compound similarity studies, where compounds in the same cluster showed synergistic effects on specific pathways. This approach helps to identify compounds that may share a common biological mechanism or therapeutic potential, providing a possible solution strategy for synergistic studies of components in Chinese medicine research. For example, various components of YXB in the cluster with blue background in Figure 4A, such as isoferulic acid, parthenolide, calyconsin and cyasterone, may have a regulatory effect on the NF-KB/IkB signaling pathway. Studies have shown that *dang gui*-derived levistilide A (Zhao et al., 2017), *dan shen*-derived protocatechualdehyde (Yang et al., 2021) and 4-coumaric acid have immunomodulatory and anti-inflammatory activities, as well as some other activities, such as antithrombotic, antioxidant, neuroprotective, etc (Yang et al., 2021; Zhu et al., 2018; Zhang et al., 2021; Li S. et al., 2020). We found that the intestinally absorbed components of YXB contain caprolactone A, protocatechualdehyde, and 4-coumaric acid, and unsupervised cluster analysis revealed that the WMs with the highest similarity in cell function to these three components were all NSAIDs. Therefore, we speculated that levistilide A, protocatechualdehyde, and 4-coumaric acid in YXB might synergistically inhibit inflammation and pain in patients. The biological process analysis in GO enrichment analysis showed that YXB may exert its medicinal effect by regulating various signals related to inflammation, pain perception and sensitivity to glucocorticoids.

Currently, the pharmacological research on YXB focuses on anti-inflammatory and analgesic aspects, and there is no research to prove that it is effective for neuropathic pain. In this study, we found that the components of YXB have a high similarity in cell function fingerprints with gabapentin, duloxetine and meperidine, which are commonly used in the treatment of neuralgia, and may be able to inhibit the NF-κB signaling pathway and pain perception (Moisset et al., 2021; Meng et al., 2019; Starnowska-Sokół and Przewłocka, 2020). In addition to regulating immunity and inflammation, the NF-κB pathway may also mediate neuropathic pain such as nerve injury, cancer neuralgia, and diabetic neuropathy pain by mediating neuroinflammation. Therefore, we speculate that YXB may have anti-neuropathic pain effects. The pain of the CCI model is close to that of human neuropathic pain, and it can mimic clinical neuropathic pain caused by various degrees of nerve compression, tumor compression, heavy metal poisoning and other factors. Through research, we found that YXB can significantly improve the pain behavior of CCI rats, and it shows a certain time-effect and dose-effect relationship. Although the analgesic efficacy of each dose of YXB on CCI models was lower than that of gabapentin, they were all superior to the non-steroidal anti-inflammatory drug indomethacin. The results of the study verified the accuracy of the predicted results of the cell function fingerprint similarity study, and found that the main mechanism of the analgesic effect of YXB on the CCI model was not through the anti-inflammatory effect.

We analyzed the anti-neuropathic pain mechanism of YXB by transcriptome research. And we found that the expression levels of Isl1, Shh and Sst were significantly downregulated in the spinal cord of CCI rats at 16 days after surgery. Shh is recognized as a key regulator of neural development, and Shh signaling has been implicated in the repair of peripheral nerve injury (Xie et al., 2022). Studies have shown that Shh is upregulated in Schwann cells adjacent to the injury site in the rat CCI model, which may promote axon regeneration and motor neuron survival after peripheral nerve injury by inducing the production of brain-derived neurotrophic factor (Hashimoto et al., 2008; Martinez et al., 2015). Isl1 is a LIM homeodomain transcription factor that plays a central role in the stages of sensory neurogenesis to differentiation, and is an essential factor for axon growth and sympathetic neuron differentiation and diversification in sympathetic nerve development, and its sustained expression is required to maintain the expression of numerous genes mediating specific sensory functions (Zhang et al., 2018; Sun et al., 2008). Sst can regulate the release of various hormones and neurotransmitters, and is an important mood and pain regulating neuropeptide (Nemes et al., 2021). Its receptor subtype 4 has analgesic, anti-inflammatory and antidepressant effects. In our study, after treatment with YXB, the expression levels of Isl1, Shh, and Sst, whose expression levels were suppressed, were significantly reversed in CCI rats. This suggests that YXB may be able to protect and promote nerve repair from nerve damage caused by sciatic nerve compression by increasing the expression of Isl1, Shh, and Sst.

Activation of ion channels is closely related to the occurrence and development of neuropathic pain, and it is also the main target of analgesic drugs (Finnerup et al., 2021). In the course of this study, we found that YXB had a down-regulating effect on a variety of upregulated ion channel-related GO items in CCI rats, such as metal ion transport, regulation of calcium ion transport, and positive regulation of ion transport items. And YXB had a significant inhibitory effect on the expression of various ion channel-related target genes in the spinal cord of CCI rats, such as Lss, Lgals3, Ugt8, Sgk1, Sgk3, Acer2 and other genes. Among them, Sgk1 can regulate the activity of various ion channels and carriers (Lou et al., 2016). The mRNA and protein expression levels of Sgk1 are increased in the spinal cord and brain of various animal models of pathological pain. Blocking the activation of Sgk1 can ameliorate allodynia induced by spinal nerve ligation in rats, as well as the development of pathological pain responses and pain caused by inflammation, nerve injury, psychiatric disorders, and chronic opioid exposure (Liu et al., 2021; Peng et al., 2013). In addition, Sgk1 is a circadian-dependent spinal cord gene that exhibits circadian oscillatory expression in the spinal cord of a mouse model of partial sciatic nerve ligation and is accompanied by rhythmic oscillations in glucocorticoid expression, leading to exacerbation of glucocorticoid-induced mechanical hyperalgesia (Koyanagi et al., 2016; Yasukochi et al., 2021). Pharmacological inhibition of Sgk1 ameliorates hyperalgesia, providing a therapeutic strategy to ameliorate nocturnal exacerbations of chronic pain (Yasukochi et al., 2021). YXB can significantly inhibit the expression of Sgk1 in the spinal cord of CCI rats, which may be one of its MoA to improve pain and hyperalgesia in CCI rats. In addition, YXB may also regulate the circadian rhythm expression of Sgk1, thereby It can improve the aggravation of pain at night in diseases such as ankylosing spondylitis, cervical spondylosis, and LDH, but this conjecture still needs further research to be verified.

This study used unsupervised cluster analysis technology to establish the similar relationship between the components contained in YXB and the pharmacological effects of WMs whose indications are the diseases and symptoms that YXB is commonly used to treat. By analyzing the pharmacological effects of WMs that closely resemble the active ingredients of YXB, it was found that in addition to anti-inflammatory and analgesic effects, YXB may also have anti-neuropathic pain properties. This may be the unique therapeutic advantage of YXB in the treatment of painful diseases. Animal experiments confirmed the accuracy of this prediction, showing that YXB can alleviate and ameliorate pain and hyperalgesia in CCI rats, and the MoA is related to its ability to regulate the expression levels of genes such as nerve damage repair and ion channels. In this study, we rapidly identified and validated the pharmacological activity of YXB in disease treatment by unsupervised learning prediction and experimental validation methods, taking into account the practical experience of YXB in treating Western diseases and symptoms over the years that it has been on the market. These results provide new research perspectives to address the challenges of elucidating the MoA of Chinese medicines and discovering appropriate clinical indications for marketed proprietary Chinese medicines.

## Data Availability

The datasets presented in this study are available in online repositories. The names of the repository/repositories and accession numbers can be found in the article/Supplementary Material. The raw data of the transcriptome sequences have been submitted to the NCBI Bioproject database (No. PRJNA1115900), available at https://www.ncbi.nlm.nih.gov/search/all/?term=PRJNA1115900. Further queries can be directed to the corresponding authors.
