# Supplementary material for: Exploring the role of Yuxuebi tablet in neuropathic pain with the method of similarity research of drug pharmacological effects based on unsupervised machine learning
Source: Front Pharmacol. 2024 Sep 17;15:1440542. doi: 10.3389/fphar.2024.1440542 (PMC11442203; doi:10.3389/fphar.2024.1440542)
Supplement: Supplementary file 1 [file DataSheet1.docx]

Supplementary Material

Exploring the role of Yuxuebi tablet in neuropathic pain with the method of similarity research of drug pharmacological effects based on unsupervised machine learning

Xiao Du^1,2#^, Chunhui Zhao^1#^, Yujie Xi^1^, Pengfei Lin^3^, Huihui Liu^3*^, Shuling Wang^2*^, Feifei Guo^1*^

*** Correspondence:** Feifei Guo: [feifei_guo@163.com](mailto:feifei_guo@163.com); Shuling Wang: wsling222@163.com; Huihui Liu: liuhui@999.com.cn

# Supplementary Data

Supplementary Material should be uploaded separately on submission. Please include any supplementary data, figures and/or tables.

Supplementary material is not typeset so please ensure that all information is clearly presented, the appropriate caption is included in the file and not in the manuscript, and that the style conforms to the rest of the article.

# Supplementary Figures and Tables

For more information on Supplementary Material and for details on the different file types accepted, please see [here](https://www.frontiersin.org/guidelines/author-guidelines#supplementary-material).

## Supplementary Figures

**9**

**1**

**3**

**4**

**8**

**6**

**5**

**7**

**2**

**8**

**7**

**2**

**1**

**6**

**5**

**4**

**3**

**9**

**Supplementary Figure 1.** HPLC-UV chromatogram (254 nm) of Yuxuebi

A.

B.

**Supplementary Figure 2.** Ion flow diagram detected by HPLC-MS/MS. (A) Determination of ion flow chart for the components in YXB’s intestinal absorption liquid; (B) Determination of ion flow chart for the components of the negative control intestinal absorption liquid.

A.


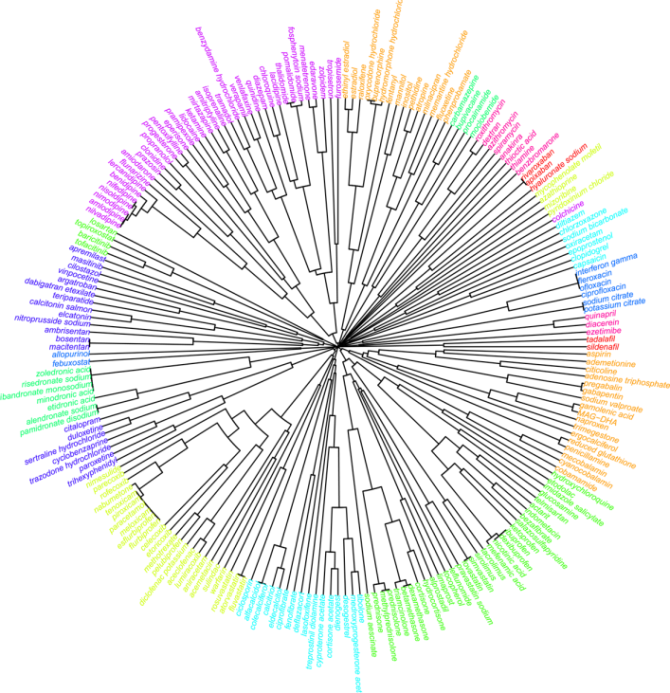


B.


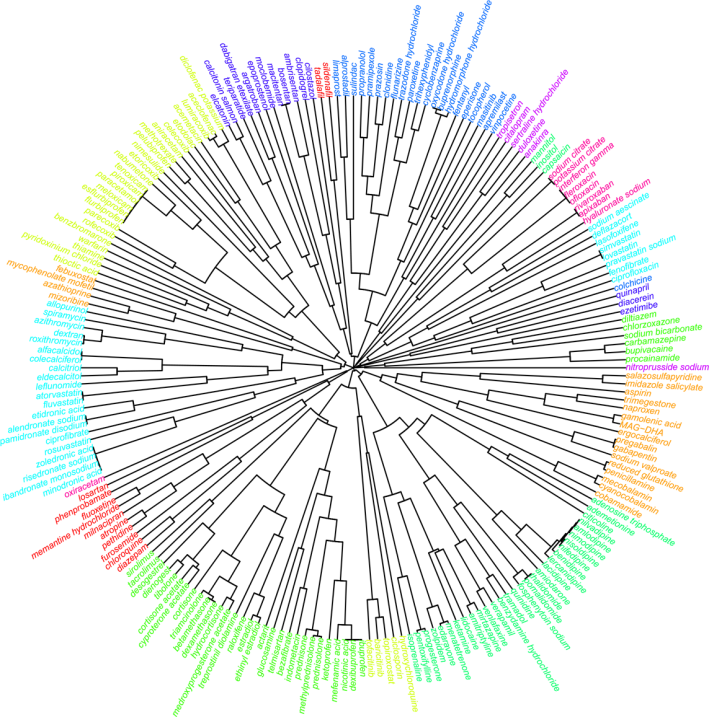


C.


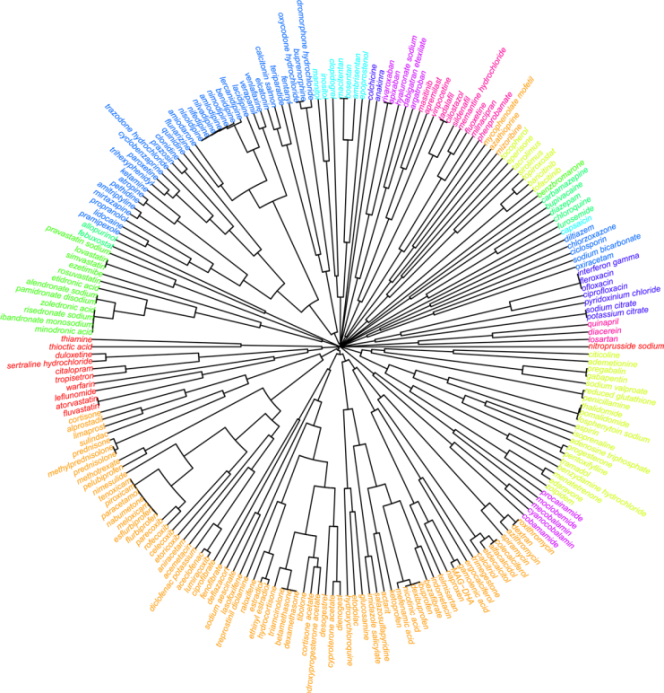


**Supplementary Figure 3.** Unsupervised hierarchical clustering of drugs based on similarity of biological functions of GO (A), REAC (B) or WP (C).

## Supplementary Tables

**Supplementary Table 1.** Information about the hospitals surveyed.

| No. | Hospital name | Hospital level | Area |
| --- | --- | --- | --- |
| 1 | Beijing Luhe Hospital Affiliated to Capital Medical University | grade III, 1st class hospital | Beijing, China |
| 2 | Dongfang Hospital Affiliated to Beijing University of Chinese Medicine | grade III, 1st class hospital | Beijing, China |
| 3 | Shunyi District Hospital of Beijing | grade III, 2nd class hospital | Beijing, China |
| 4 | Shunyi District of Beijing Ditan Hospital | grade III, 1st class hospital | Beijing, China |
| 5 | Guang'anmen Hospital,China Academy of Chinese Medical Sciences | grade III, 1st class hospital | Beijing, China |
| 6 | Wangjing Hospital of China Academy of Chinese Medical Sciences | grade III, 1st class hospital | Beijing, China |
| 7 | Beijing Dongcheng District People’s Hospital | grade II, 1st class hospital | Beijing, China |
| 8 | Xinhua Hospital Affiliated to Shanghai Jiaotong University School of Medicine Chongming Branch | grade III, 2nd class hospital | Shanghai, China |
| 9 | Shanghai Fengxian District Central Hospital | grade III, 2nd class hospital | Shanghai, China |
| 10 | Qingpu Branch of Zhongshan Hospital Affiliated to Fudan University | grade III, 2nd class hospital | Shanghai, China |
| 11 | Minhang District Central Hospital | grade II, 1st class hospital | Shanghai, China |
| 12 | Shanghai Pudong New Area Gongli Hospital | grade II, 1st class hospital | Shanghai, China |
| 13 | Shanghai Qingpu District Hospital of Traditional Chinese Medicine | grade II, 1st class hospital | Shanghai, China |
| 14 | Shanghai Putuo District People’s Hospital | grade II, 1st class hospital | Shanghai, China |
| 15 | Community Health Service in Gangxi Town of Shanghai | Community hospital | Shanghai, China |
| 16 | Community Health Service in Chengqiao Town of Chongming District, Shanghai | Community hospital | Shanghai, China |

**Supplementary Table 2.** The distribution of these doctors' departments

| No. | Department name | Number of respondents |
| --- | --- | --- |
| 1 | Orthopaedics | 28 |
| 2 | traditional Chinese medicine | 6 |
| 3 | rheumatism and immunology | 6 |
| 4 | general practice | 5 |
| 5 | disease prevention and health care | 3 |
| 6 | massage department | 3 |
| 7 | geriatrics department | 2 |
| 8 | Internal Medicine department | 2 |
| 9 | pain department | 1 |
| 10 | rehabilitation department | 1 |
| 11 | cadre department | 1 |
| 12 | traumatological department of TCM | 1 |
| 13 | General Surgery | 1 |
| 14 | Vascular Surgery | 1 |
| 15 | Ophthalmology | 1 |
| 16 | neurosurgery | 1 |
| 17 | Department of sports medicine | 1 |

**Supplementary Table 3.** The components identified by UPLC-MS/MS in YXB.

| No. | detected Mass (Da) | tR (min) | Area (Average) | Identification | Molecular formula |
| --- | --- | --- | --- | --- | --- |
| 1 | 216.1516 | 32.03 | 152162840 | (+)-ar-Turmerone | C_15_H_20_O |
| 2 | 341.1630 | 19.45 | 390351601 | (+)-Magnoflorine | C_20_H_23_NO_4_ |
| 3 | 254.1519 | 30.32 | 26648147 | (+/-)-C75 | C_14_H_22_O_4_ |
| 4 | 314.2458 | 35.68 | 153381283 | (+/-)12(13)-DiHOME | C_18_H_34_O_4_ |
| 5 | 220.1828 | 30.06 | 63344141 | (-)-Caryophyllene oxide | C_15_H_24_O |
| 6 | 310.2145 | 37.61 | 23924243 | (11E,15Z)-9,10,13-trihydroxyoctadeca-11,15-dienoic acid | C_18_H_32_O_5_ |
| 7 | 330.2406 | 28.47 | 1984147404 | (15Z)-9,12,13-Trihydroxy-15-octadecenoic acid | C_18_H_34_O_5_ |
| 8 | 308.1049 | 27.58 | 66663543 | (1E,4Z,6E)-5-hydroxy-1,7-bis(4-hydroxyphenyl)hepta-1,4,6-trien-3-one | C_19_H_16_O_4_ |
| 9 | 286.0843 | 27.05 | 20173508 | (1R,10R)-5-methoxy-8,17-dioxatetracyclo[8.7.0.0Â?,â·.0ÂaÂa,Âaâ¶]heptadeca-2,4,6,11(16),12,14-hexaene-6,14-diol | C_16_H_14_O_5_ |
| 10 | 452.3293 | 35.86 | 38734697 | (1R,2R,5S,8R,10R,14R)-20-hydroxy-1,2,14,18,18-pentamethyl-17-oxo-8-(prop-1-en-2-yl)pentacyclo[11.8.0.0Â?,Âaâ°.0âµ,âa.0Âaâ´,Âaâa]henicosane-5-carboxylic acid | C_30_H_46_O_4_ |
| 11 | 232.1464 | 35.02 | 43974958 | (1R,2R,6R,9R)-2,11,11-trimethyl-3-oxotricyclo[4.3.2.0Âa,âµ]undecane-9-carboxylic acid | C_15_H_22_O_3_ |
| 12 | 352.2252 | 26.03 | 25966982 | (1R,2S,3R,5S,6R,8aR)-5-[2-(furan-3-yl)ethyl]-5,6,8a-trimethyl-octahydro-2H-spiro[naphthalene-1,2'-oxirane]-2,3-diol | C_20_H_30_O_4_ |
| 13 | 306.2558 | 46.55 | 2670419371 | (1S,2S,4aR,7R,8aR)-1-[(3Z)-5-hydroxy-3-methylpent-3-en-1-yl]-2,5,5,8a-tetramethyl-decahydronaphthalene-2,7-diol | C_20_H_36_O_3_ |
| 14 | 290.2610 | 48.57 | 2060282663 | (1S,2S,8aR)-1-[(3Z)-5-hydroxy-3-methylpent-3-en-1-yl]-2,5,5,8a-tetramethyl-decahydronaphthalen-2-ol | C_20_H_36_O_2_ |
| 15 | 724.4039 | 28.94 | 8750174 | (1S,2S,8ξ,9β,16α,17ξ)-1-(β-D-Glucopyranosyloxy)-2,16,20-trihydroxy-9,10,14-trimethyl-11,22-dioxo-4,9-cyclo-9,10-secocholest-5-en-25-yl acetate | C_38_H_60_O_13_ |
| 16 | 454.3449 | 28.09 | 42544330 | (1S,4S,5R,10S,13S,17S,19S,20R)-10-hydroxy-4,5,9,9,13,19,20-heptamethyl-24-oxahexacyclo[15.5.2.0Âa,Âaâ¸.0â´,Âaâ·.0âµ,Âaâ´.0â¸,ÂaÂl]tetracos-15-en-23-one | C_30_H_46_O_3_ |
| 17 | 300.2089 | 50.65 | 12345120 | (1S,4S,5R,9R,13S)-5,9-dimethyl-14-methylidenetetracyclo[11.2.1.0Âa,Âaâ°.0â´,âa]hexadec-10-ene-5-carboxylic acid | C_20_H_28_O_2_ |
| 18 | 264.1363 | 23.60 | 27553389 | (1S,5R,9R,13R)-1,5,9-trimethyl-11,14,15,16-tetraoxatetracyclo[10.3.1.0â´,ÂaÂl.0â¸,ÂaÂl]hexadecan-10-one | C_15_H_22_O_5_ |
| 19 | 450.1166 | 20.01 | 52989741 | (1ξ)-1,5-Anhydro-1-[(2R,3R)-3,5,7-trihydroxy-2-(4-hydroxyphenyl)-4-oxo-3,4-dihydro-2H-chromen-6-yl]-D-glucitol | C_21_H_22_O_11_ |
| 20 | 280.1311 | 21.44 | 44834279 | (2E)-2-(hydroxymethyl)-3-[3-oxo-5-(propan-2-yl)-1,3,4,5,6,7-hexahydro-2-benzofuran-4-yl]prop-2-enoic acid | C_15_H_20_O_5_ |
| 21 | 316.2040 | 45.58 | 21818260 | (2E)-2-[2-(1,2,4a,5-tetramethyl-1,2,3,4,4a,7,8,8a-octahydronaphthalen-1-yl)ethyl]but-2-enedioic acid | C_20_H_30_O_4_ |
| 22 | 308.0895 | 23.87 | 126634832 | (2E)-3-(2-{[(2S,3R,4S,5S,6R)-3,4,5-trihydroxy-6-(hydroxymethyl)oxan-2-yl]oxy}phenyl)prop-2-enoic acid | C_15_H_18_O_8_ |
| 23 | 300.2091 | 46.30 | 63261466 | (2E)-5-[(8aS)-2,5,5,8a-tetramethyl-3-oxo-3,4,4a,5,6,7,8,8a-octahydronaphthalen-1-yl]-3-methylpent-2-enoic acid | C_20_H_30_O_3_ |
| 24 | 452.3294 | 41.53 | 162849405 | (2E,6S)-7-hydroxy-2-methyl-6-[(2S,11S,14S,15S)-2,6,6,11,15-pentamethyl-5-oxotetracyclo[8.7.0.0Â?,â·.0ÂaÂa,Âaâµ]heptadec-1(10)-en-14-yl]hept-2-enoic acid | C_30_H_46_O_4_ |
| 25 | 718.1536 | 23.83 | 7475857325 | (2R)-2-({(2E)-3-[3-{[(1R)-1-Carboxy-2-(3,4-dihydroxyphenyl)ethoxy]carbonyl}-2-(3,4-dihydroxyphenyl)-7-hydroxy-2,3-dihydro-1-benzofuran-4-yl]-2-propenoyl}oxy)-3-(3,4-dihydroxyphenyl)propanoic acid | C_36_H_30_O_16_ |
| 26 | 246.0893 | 30.45 | 54298613 | (2S)-2-(2-hydroxypropan-2-yl)-2H,3H,7H-furo[3,2-g]chromen-7-one | C_14_H_14_O_4_ |
| 27 | 470.3400 | 40.84 | 58171749 | (2S,4aS,6aS,6bR,10S,12aS,14bS)-10-hydroxy-2,4a,6a,6b,9,9,12a-heptamethyl-13-oxo-1,2,3,4,4a,5,6,6a,6b,7,8,8a,9,10,11,12,12a,12b,13,14b-icosahydropicene-2-carboxylic acid | C_30_H_46_O_4_ |
| 28 | 300.2090 | 40.82 | 655751368 | (2Z)-5-(1,2,4a,5-tetramethyl-7-oxo-1,2,3,4,4a,7,8,8a-octahydronaphthalen-1-yl)-3-methylpent-2-enoic acid | C_20_H_30_O_3_ |
| 29 | 488.3504 | 35.33 | 68927214 | (2α,3β,19α)-2,3,19-Trihydroxyolean-12-en-28-oic acid | C_30_H_48_O_5_ |
| 30 | 246.1256 | 29.14 | 137664538 | (3aR,7aS,8S,9aR)-5,8-dimethyl-3-methylidene-2H,3H,3aH,4H,6H,7H,7aH,8H,9H,9aH-azuleno[6,5-b]furan-2,6-dione | C_15_H_18_O_3_ |
| 31 | 956.4985 | 33.30 | 8235902 | (3beta,5xi,9xi,22beta)-22,24-Dihydroxyolean-12-en-3-yl 6-deoxy-alpha-L-mannopyranosyl-(1-2)-beta-D-galactopyranuronosyl-(1-2)-beta-D-glucopyranosiduronic acid | C_48_H_76_O_19_ |
| 32 | 502.2933 | 22.81 | 2167320842 | (3S,4S,5R)-4-[(2R,3R)-2,3-dihydroxy-3-[(2R,4S,5R,7R,11S,14S,15R)-4,5,11-trihydroxy-2,15-dimethyl-8-oxotetracyclo[8.7.0.0Â?,â·.0ÂaÂa,Âaâµ]heptadec-9-en-14-yl]butyl]-3,5-dimethyloxolan-2-one | C_29_H_44_O_8_ |
| 33 | 796.4614 | 32.04 | 5511433 | (3β,5ξ,9ξ)-3-{[2-O-(β-D-Glucopyranosyl)-β-D-glucopyranosyl]oxy}-23-hydroxyolean-12-en-28-oic acid | C_42_H_68_O_14_ |
| 34 | 940.5039 | 33.29 | 79465607 | (3β,5ξ,9ξ,18ξ)-28-Hydroxy-28-oxoolean-12-en-3-yl 6-deoxy-α-L-mannopyranosyl-(1->3)-[β-D-glucopyranosyl-(1->2)]-β-D-glucopyranosiduronic acid | C_48_H_76_O_18_ |
| 35 | 214.1360 | 39.18 | 127365176 | (4aR,5R,6R)-6-hydroxy-4a,5-dimethyl-3-(prop-1-en-2-yl)-2,4a,5,6,7,8-hexahydronaphthalen-2-one | C_15_H_20_O_2_ |
| 36 | 276.0999 | 26.11 | 8808086 | (4S,5R,8R,12R)-12-hydroxy-12-(hydroxymethyl)-3,4-dimethyl-11-oxo-10-oxatricyclo[6.4.0.0Âa,âµ]dodeca-2,6-diene-7-carboxylic acid | C_15_H_18_O_6_ |
| 37 | 284.2141 | 32.34 | 43584861 | (9cis)-Retinal | C_20_H_28_O |
| 38 | 244.2037 | 40.65 | 16034151 | (R)-3-Hydroxy myristic acid | C_14_H_28_O_3_ |
| 39 | 122.1096 | 48.57 | 36293730 | 1,2,3,4-Tetramethyl-1,3-cyclopentadiene | C_9_H_14_ |
| 40 | 368.1108 | 20.62 | 37339423 | 1,3,5-trihydroxy-4-{[(2E)-3-(3-hydroxy-4-methoxyphenyl)prop-2-enoyl]oxy}cyclohexane-1-carboxylic acid | C_17_H_20_O_9_ |
| 41 | 516.1271 | 20.20 | 27316889 | 1,3-Dicaffeoylquinic acid | C_25_H_24_O_12_ |
| 42 | 252.1726 | 24.30 | 246138129 | 1,4-dihydroxy-1,4-dimethyl-7-(propan-2-ylidene)-decahydroazulen-6-one | C_15_H_24_O_3_ |
| 43 | 316.1674 | 24.77 | 8204086 | 1,7-Bis(4-hydroxyphenyl)-3,5-heptanediol | C_19_H_24_O_4_ |
| 44 | 266.1518 | 24.82 | 11748299 | 1,9b-Dihydroxy-6,6,9a-trimethyl-5,5a,6,7,8,9,9a,9b-octahydronaphtho[1,2-c]furan-3(1H)-one | C_15_H_22_O_4_ |
| 45 | 340.0583 | 23.84 | 157716176 | 1-(3,4-dihydroxyphenyl)-6,7-dihydroxy-1,2-dihydronaphthalene-2,3-dicarboxylic acid | C_18_H_14_O_8_ |
| 46 | 186.0892 | 20.45 | 6620173 | 1-(Carboxymethyl)cyclohexanecarboxylic acid | C_9_H_14_O_4_ |
| 47 | 360.1422 | 21.11 | 19803722 | 1-(β-D-Glucopyranosyloxy)-7-methyl-1,4a,5,6,7,7a-hexahydrocyclopenta[c]pyran-4-carboxylic acid | C_16_H_24_O_9_ |
| 48 | 308.1049 | 36.05 | 142032928 | 1-[2-(1,3-Benzodioxol-5-yl)-3-methyl-1-benzofuran-5-yl]-1,2-propanediol | C_19_H_18_O_5_ |
| 49 | 354.0953 | 18.18 | 246139758 | 1-Caffeoylquinic acid | C_16_H_18_O_9_ |
| 50 | 336.2667 | 43.07 | 23814957 | 1-Linoleoyl glycerol | C_21_H_38_O_4_ |
| 51 | 307.2149 | 25.53 | 71415812 | 10-Nitrolinoleate | C_18_H_31_NO_4_ |
| 52 | 322.2510 | 40.95 | 455992576 | 11-Deoxy prostaglandin F1β | C_20_H_36_O_4_ |
| 53 | 292.2039 | 33.45 | 104930275 | 12-oxo Phytodienoic Acid | C_18_H_28_O_3_ |
| 54 | 356.2564 | 30.38 | 63213390 | 13,14-dihydro Prostaglandin E1 | C_20_H_36_O_5_ |
| 55 | 512.3505 | 45.13 | 18010154 | 13-hydroxy-2,6,6,10,11-pentamethyl-14-(4,5,6-trihydroxy-6-methylheptan-2-yl)tetracyclo[8.7.0.0Â?,â·.0ÂaÂa,Âaâµ]heptadec-14-en-5-one | C_30_H_50_O_5_ |
| 56 | 318.1469 | 26.26 | 18990832 | 14,16-dihydroxy-3-methyl-3,4,5,6,7,8,9,10-octahydro-1H-2-benzoxacyclotetradecine-1,7-dione | C_18_H_22_O_5_ |
| 57 | 352.2249 | 50.19 | 70020143 | 15-keto Prostaglandin E1 | C_20_H_32_O_5_ |
| 58 | 334.2145 | 33.59 | 21191675 | 15-keto Prostaglandin F2? | C_20_H_32_O_5_ |
| 59 | 346.2509 | 45.79 | 34127159 | 16,16-Dimethyl prostaglandin A1 | C_22_H_36_O_4_ |
| 60 | 272.2352 | 45.97 | 196452191 | 16-Hydroxyhexadecanoic acid | C_16_H_32_O_3_ |
| 61 | 286.1571 | 42.70 | 304130185 | 16α-Hydroxyestrone | C_18_H_22_O_3_ |
| 62 | 272.1777 | 37.36 | 117035583 | 17?-Estradiol | C_18_H_24_O_2_ |
| 63 | 470.3399 | 39.99 | 43796312 | 18-β-Glycyrrhetinic acid | C_30_H_46_O_4_ |
| 64 | 332.1988 | 32.77 | 28713599 | 19(R)-hydroxy Prostaglandin A2 | C_20_H_30_O_5_ |
| 65 | 352.2238 | 29.81 | 56313466 | 19(R)-hydroxy Prostaglandin E1 | C_20_H_34_O_6_ |
| 66 | 216.0901 | 17.99 | 37208948 | 2,3,4,9-Tetrahydro-1H-β-carboline-3-carboxylic acid | C_12_H_12_N_2_O_2_ |
| 67 | 154.0266 | 16.50 | 36549080 | 2,3-Dihydroxybenzoic acid | C_7_H_6_O_4_ |
| 68 | 288.0632 | 23.73 | 15863401 | 2,4,6-Trihydroxy-2-(4-hydroxybenzyl)-1-benzofuran-3(2H)-one | C_15_H_12_O_6_ |
| 69 | 168.0423 | 1.89 | 6514119 | 2,4,6-Trihydroxyacetophenone | C_8_H_8_O_4_ |
| 70 | 134.0732 | 34.26 | 14768933 | 2,4-Dimethylbenzaldehyde | C_9_H_10_O |
| 71 | 432.1633 | 17.24 | 3487523 | 2-(4-Hydroxyphenyl)ethyl 6-O-[(2R,3R,4R)-3,4-dihydroxy-4-(hydroxymethyl)tetrahydro-2-furanyl]-beta-D-glucopyranoside | C_19_H_28_O_11_ |
| 72 | 462.2468 | 20.81 | 20385081 | 2-(4-Methyl-3-cyclohexen-1-yl)-2-propanyl 6-O-(6-deoxy-α-L-mannopyranosyl)-β-D-glucopyranoside | C_22_H_38_O_10_ |
| 73 | 274.1545 | 27.43 | 14802167 | 2-(8-Hydroxy-4a,8-dimethyldecahydro-2-naphthalenyl)acrylic acid | C_15_H_24_O_3_ |
| 74 | 250.1568 | 31.79 | 73171445 | 2-[(2S,4aR,8aS)-2-Hydroxy-4a-methyl-8-methylenedecahydro-2-naphthalenyl]acrylic acid | C_15_H_22_O_3_ |
| 75 | 344.1626 | 23.59 | 14319015 | 2-[1-(2H-1,3-benzodioxol-5-yl)propan-2-yl]-6-methoxy-4-(prop-2-en-1-yl)phenol | C_20_H_22_O_4_ |
| 76 | 344.1472 | 19.64 | 9946663 | 2-Hydroxy-1-(4-methoxyphenyl)propyl hexopyranoside | C_16_H_24_O_8_ |
| 77 | 152.0475 | 21.21 | 88765926 | 2-Hydroxy-4-methoxybenzaldehyde | C_8_H_8_O_3_ |
| 78 | 342.2172 | 48.53 | 18523666 | 2-hydroxy-6-[(8Z,11Z)-pentadeca-8,11,14-trien-1-yl]benzoic acid | C_22_H_30_O_3_ |
| 79 | 124.0524 | 19.39 | 55554670 | 2-Hydroxybenzyl alcohol | C_7_H_8_O_2_ |
| 80 | 132.0786 | 19.90 | 99015449 | 2-Hydroxycaproic acid | C_6_H_12_O_3_ |
| 81 | 146.0369 | 29.56 | 24305319 | 2-Hydroxycinnamic acid | C_9_H_8_O_3_ |
| 82 | 176.0685 | 18.45 | 31591742 | 2-Isopropylmalic acid | C_7_H_12_O_5_ |
| 83 | 136.0525 | 16.56 | 82761904 | 2-Methylbenzoic acid | C_8_H_8_O_2_ |
| 84 | 144.0575 | 33.75 | 46050430 | 2-Naphthol | C_10_H_8_O |
| 85 | 151.0635 | 1.70 | 13605252 | 2-Phenylglycine | C_8_H_9_NO_2_ |
| 86 | 330.1832 | 41.93 | 10717418 | 3,3'-Diisopropyl-6,6'-dimethyl-2,2',5,5'-biphenyltetrol | C_20_H_26_O_4_ |
| 87 | 302.1269 | 17.93 | 73882597 | 3,4-dihydroxy-4-(4-methoxyphenyl)-1,2,3,4-tetrahydroquinolin-2-one | C_16_H_15_NO_4_ |
| 88 | 154.0630 | 16.92 | 4352013 | 3,4-Dihydroxyphenylethanol | C_8_H_10_O_3_ |
| 89 | 182.0580 | 19.11 | 12962441 | 3,4-Dihydroxyphenylpropionic acid | C_9_H_10_O_4_ |
| 90 | 516.1269 | 22.52 | 282843450 | 3,5-Dicaffeoylquinic acid | C_25_H_24_O_12_ |
| 91 | 182.0588 | 26.01 | 15453722 | 3,5-Dimethoxy-4-hydroxybenzaldehyde | C_9_H_10_O_4_ |
| 92 | 196.1214 | 18.91 | 8437628 | 3-(propan-2-yl)-octahydropyrrolo[1,2-a]pyrazine-1,4-dione | C_10_H_16_N_2_O_2_ |
| 93 | 358.1265 | 18.02 | 9819113 | 3-[2-(β-D-Glucopyranosyloxy)-4-methoxyphenyl]propanoic acid | C_16_H_22_O_9_ |
| 94 | 358.1265 | 19.01 | 14220623 | 3-[3-(beta-D-Glucopyranosyloxy)-2-methoxyphenyl]propanoic acid | C_16_H_22_O_9_ |
| 95 | 188.0838 | 29.56 | 174862317 | 3-Butylidenephthalide | C_12_H_12_O_2_ |
| 96 | 109.0528 | 1.85 | 201975186 | 3-Hydroxy-2-methylpyridine | C_6_H_7_NO |
| 97 | 268.0736 | 24.72 | 435986218 | 3-hydroxy-6-methoxy-2-phenyl-4H-chromen-4-one | C_16_H_12_O_4_ |
| 98 | 138.0316 | 23.58 | 73962276 | 3-Hydroxybenzoic acid | C_7_H_6_O_3_ |
| 99 | 124.0524 | 17.64 | 8309323 | 3-Hydroxybenzyl alcohol | C_7_H_8_O_2_ |
| 100 | 168.0423 | 19.39 | 299812265 | 3-Hydroxymandelic acid | C_8_H_8_O_4_ |
| 101 | 456.3606 | 51.14 | 80373758 | 3-Hydroxyurs-12-en-23-oic acid | C_30_H_48_O_3_ |
| 102 | 298.1935 | 49.07 | 37908124 | 3-methoxy-1,13-dimethyl-9,11,12,13,14,15,16,17-octahydro-8H-cyclopenta[a]phenanthren-17-ol | C_20_H_26_O_2_ |
| 103 | 136.0525 | 33.97 | 49264947 | 3-Methoxybenzaldehyde | C_8_H_8_O_2_ |
| 104 | 168.0421 | 26.22 | 31235931 | 3-Methoxysalicylic acid | C_8_H_8_O_4_ |
| 105 | 348.2665 | 50.21 | 129084964 | 3-Methyl-5-(5,5,8a-trimethyl-2-methylene-7-oxodecahydro-1-naphthalenyl)pentyl acetate | C_22_H_36_O_3_ |
| 106 | 114.0470 | 46.54 | 19199999 | 3-Phenylpropionitrile | C_9_H_9_N |
| 107 | 202.1205 | 25.20 | 45331355 | 3-tert-Butyladipic acid | C_10_H_18_O_4_ |
| 108 | 248.1412 | 35.05 | 42021460 | 3a,8-dihydroxy-3,5a,9-trimethyl-2H,3H,3aH,4H,5H,5aH,6H,7H,8H,9bH-naphtho[1,2-b]furan-2-one | C_15_H_22_O_4_ |
| 109 | 262.1207 | 26.37 | 70990691 | 4,6-dihydroxy-5a-methyl-3-methylidene-2-oxo-dodecahydronaphtho[1,2-b]furan-9-carbaldehyde | C_15_H_20_O_5_ |
| 110 | 124.0638 | 1.85 | 183412470 | 4,6-Dimethyl-2(1H)-pyrimidinone | C_6_H_8_N_2_O |
| 111 | 268.1676 | 38.39 | 5449243 | 4,7-dihydroxy-4-(hydroxymethyl)-3,4a,8,8-tetramethyl-1,4,4a,5,6,7,8,8a-octahydronaphthalen-1-one | C_15_H_24_O_4_ |
| 112 | 248.1413 | 30.71 | 155762811 | 4,8-dihydroxy-6,6,8-trimethyl-1H,3H,4H,4aH,5H,6H,7H,7aH,8H,9H-azuleno[5,6-c]furan-1-one | C_15_H_22_O_4_ |
| 113 | 204.0788 | 26.90 | 11178652 | 4-(2,3-dihydro-1,4-benzodioxin-6-yl)butanoic acid | C_12_H_14_O_4_ |
| 114 | 374.1577 | 19.86 | 31972467 | 4-(3-Hydroxybutyl)phenyl β-D-glucopyranoside | C_16_H_24_O_7_ |
| 115 | 222.0892 | 25.90 | 58940395 | 4-(4-Ethoxyphenyl)-4-oxobutanoic acid | C_12_H_14_O_4_ |
| 116 | 340.1313 | 26.13 | 69740435 | 4-[(1S,3aR,4S,6aR)-4-(4-hydroxy-3-methoxyphenyl)-hexahydrofuro[3,4-c]furan-1-yl]-2-methoxyphenol | C_20_H_22_O_6_ |
| 117 | 109.0529 | 3.51 | 24030791 | 4-Aminophenol | C_6_H_7_NO |
| 118 | 164.0475 | 21.33 | 72514324 | 4-Coumaric acid | C_9_H_8_O_3_ |
| 119 | 326.1915 | 43.84 | 98203406 | 4-Dodecylbenzenesulfonic acid | C_18_H_30_O_3_S |
| 120 | 152.0473 | 19.18 | 6952178 | 4-Hydroxyphenylacetic acid | C_8_H_8_O_3_ |
| 121 | 192.0788 | 24.08 | 91363391 | 4-methoxy-6-(prop-2-en-1-yl)-2H-1,3-benzodioxole | C_11_H_12_O_3_ |
| 122 | 155.0218 | 21.35 | 10410439 | 4-Nitrocatechol | C_6_H_5_NO_4_ |
| 123 | 129.0427 | 1.86 | 1332444372 | 4-Oxoproline | C_5_H_7_NO_3_ |
| 124 | 338.1508 | 43.00 | 3075242 | 4-{3-[(4-hydroxy-3-methoxyphenyl)methyl]-2-methylbutyl}benzene-1,2-diol | C_19_H_24_O_4_ |
| 125 | 312.1361 | 32.40 | 79424880 | 5'-(furan-3-yl)-4a-hydroxy-2,5-dimethyl-3,4,4a,7,8,8a-hexahydro-2H-spiro[naphthalene-1,3'-oxolane]-2',7-dione | C_19_H_22_O_5_ |
| 126 | 318.2196 | 33.84 | 43825269 | 5(S),15(S)-DiHETE | C_20_H_32_O_4_ |
| 127 | 594.1590 | 22.14 | 97772943 | 5,7-Dihydroxy-2-(4-hydroxyphenyl)-4-oxo-4H-chromen-3-yl 6-O-(6-deoxyhexopyranosyl)hexopyranoside | C_27_H_30_O_15_ |
| 128 | 314.0791 | 26.03 | 103277882 | 5,7-dihydroxy-3,8-dimethoxy-2-phenyl-4H-chromen-4-one | C_17_H_14_O_6_ |
| 129 | 320.2351 | 52.45 | 344453886 | 5-(1,2,4a,5-tetramethyl-7-oxo-1,2,3,4,4a,7,8,8a-octahydronaphthalen-1-yl)-3-methylpentanoic acid | C_20_H_32_O_3_ |
| 130 | 226.1570 | 22.45 | 21883317 | 5-(6-hydroxy-6-methyloctyl)-2,5-dihydrofuran-2-one | C_13_H_22_O_3_ |
| 131 | 210.1257 | 23.35 | 35684111 | 5-[(1E)-3-hydroxy-3-methylbut-1-en-1-yl]-2-methylcyclohex-5-ene-1,2,4-triol | C_12_H_20_O_4_ |
| 132 | 350.2457 | 38.84 | 30105566 | 5-[5-(methoxycarbonyl)-5,8a-dimethyl-2-methylidene-decahydronaphthalen-1-yl]-3-methylpentanoic acid | C_21_H_34_O_4_ |
| 133 | 354.2407 | 33.84 | 98581247 | 5-[5-hydroxy-3-(hydroxymethyl)pentyl]-8a-(hydroxymethyl)-5,6-dimethyl-3,4,4a,5,6,7,8,8a-octahydronaphthalene-1-carboxylic acid | C_20_H_34_O_5_ |
| 134 | 144.0425 | 1.96 | 137044312 | 5-hydroxy-4-methoxy-5,6-dihydro-2H-pyran-2-one | C_6_H_8_O_4_ |
| 135 | 126.0318 | 1.54 | 137710636 | 5-Hydroxymethyl-2-furaldehyde | C_6_H_6_O_3_ |
| 136 | 338.1004 | 27.41 | 16784974 | 5-methyl-4-{[(2S,3R,4S,5S,6R)-3,4,5-trihydroxy-6-(hydroxymethyl)oxan-2-yl]oxy}-2H-chromen-2-one | C_16_H_18_O_8_ |
| 137 | 125.0591 | 1.86 | 52550475 | 5-Methylcytosine | C_5_H_7_N_3_O |
| 138 | 488.1321 | 23.76 | 67905440 | 6''-O-Acetylglycitin | C_24_H_24_O_11_ |
| 139 | 210.0793 | 30.31 | 17109653 | 6,7-dihydro-5H-dibenzo[d,f][1,3]diazepin-6-one | C_13_H_10_N_2_O |
| 140 | 188.0473 | 33.75 | 16420621 | 6-Hydroxy-2-naphthoic acid | C_11_H_8_O_3_ |
| 141 | 250.1570 | 31.11 | 143321842 | 6-hydroxy-3,5a,9-trimethyl-2H,3H,3aH,4H,5H,5aH,6H,7H,9aH,9bH-naphtho[1,2-b]furan-2-one | C_15_H_22_O_3_ |
| 142 | 928.5032 | 26.78 | 2923063178 | 6-O-Hexopyranosyl-1-O-[19-hydroxy-28-oxo-3-(pentopyranosyloxy)olean-12-en-28-yl]hexopyranose | C_47_H_76_O_18_ |
| 143 | 326.1155 | 34.49 | 517286035 | 6a,7,8,12a-tetrahydroxy-3-methyl-1,2,3,4,5,6,6a,7,12,12a-decahydrotetraphene-1,12-dione | C_19_H_20_O_6_ |
| 144 | 312.1362 | 22.16 | 7402705 | 7-(3,4-dihydroxyphenyl)-5-hydroxy-1-(4-hydroxyphenyl)heptan-3-one | C_19_H_22_O_5_ |
| 145 | 192.0424 | 21.99 | 50226325 | 7-hydroxy-6-methoxy-2H-chromen-2-one | C_10_H_8_O_4_ |
| 146 | 302.0792 | 25.08 | 15896481 | 7-Methoxy-5,3',4'-trihydroxyflavanone | C_16_H_14_O_6_ |
| 147 | 338.2458 | 24.55 | 66799291 | 8-iso Prostaglandin F1? | C_20_H_36_O_5_ |
| 148 | 232.1464 | 34.26 | 49657750 | 9-hydroxy-2,10,10-trimethyltricyclo[6.3.0.0Âa,âµ]undec-6-ene-6-carboxylic acid | C_15_H_22_O_3_ |
| 149 | 294.2196 | 42.50 | 529423810 | 9-Oxo-10(E),12(E)-octadecadienoic acid | C_18_H_30_O_3_ |
| 150 | 120.0576 | 1.87 | 36775820 | Acetophenone | C_8_H_8_O |
| 151 | 512.3504 | 48.00 | 5734675041 | Acetyl-11-keto-β-boswellic acid | C_32_H_48_O_5_ |
| 152 | 526.1689 | 20.67 | 10416582 | albiflorin | C_23_H_28_O_11_ |
| 153 | 528.3458 | 45.97 | 42247237 | Alisol C 23-acetate | C_32_H_48_O_6_ |
| 154 | 158.0440 | 1.62 | 209747194 | Allantoin | C_4_H_6_N_4_O_3_ |
| 155 | 270.0529 | 22.15 | 18710792 | Apigenin | C_15_H_10_O_5_ |
| 156 | 286.2297 | 43.78 | 119740676 | Arachidonic acid | C_20_H_32_O_2_ |
| 157 | 303.2563 | 52.47 | 17099992 | Arachidonoyl amide | C_20_H_33_NO |
| 158 | 448.1008 | 21.83 | 16083188 | Astragalin | C_21_H_20_O_11_ |
| 159 | 830.4669 | 29.09 | 238821638 | Astragaloside IV | C_41_H_68_O_14_ |
| 160 | 230.1308 | 36.53 | 215698587 | Atractylenolide I | C_15_H_18_O_2_ |
| 161 | 188.1048 | 23.22 | 379290634 | Azelaic acid | C_9_H_16_O_4_ |
| 162 | 328.0796 | 17.39 | 4644353 | Bengenin | C_14_H_16_O_9_ |
| 163 | 122.0367 | 20.00 | 296347204 | Benzoic acid | C_7_H_6_O_2_ |
| 164 | 117.0790 | 1.56 | 4232971014 | Betaine | C_5_H_11_NO_2_ |
| 165 | 308.1049 | 33.75 | 415148223 | Bisdemethoxycurcumin | C_19_H_16_O_4_ |
| 166 | 337.3345 | 48.68 | 510368412 | Bitertanol | C_20_H_23_N_3_O_2_ |
| 167 | 248.1778 | 48.13 | 389666223 | Bryodulcosigenin | C_30_H_50_O_4_ |
| 168 | 148.0890 | 19.22 | 7302672 | Butyrophenone | C_10_H_12_O |
| 169 | 180.0423 | 23.22 | 3407127599 | Caffeic acid | C_9_H_8_O_4_ |
| 170 | 284.0685 | 25.63 | 3113550345 | Calycosin | C_16_H_12_O_5_ |
| 171 | 202.5476 | 21.42 | 211828084 | Calycosin-7-O-β-D-glucoside | C_22_H_22_O_10_ |
| 172 | 196.0738 | 19.24 | 69320762 | Cantharidin | C_10_H_12_O_4_ |
| 173 | 354.0953 | 19.06 | 239651510 | Chlorogenic acid | C_16_H_18_O_9_ |
| 174 | 103.0997 | 1.48 | 2457907891 | Choline | C_5_H_13_NO |
| 175 | 306.1104 | 22.00 | 65904128 | Cimifugin | C_16_H_18_O_6_ |
| 176 | 148.0526 | 25.04 | 11066575 | Cinnamic acid | C_9_H_8_O_2_ |
| 177 | 346.2510 | 43.81 | 49785116 | Ciprostene | C_22_H_36_O_4_ |
| 178 | 192.0270 | 3.59 | 6028669 | Citric acid | C_6_H_8_O_7_ |
| 179 | 328.2250 | 27.27 | 302164241 | Corchorifatty acid F | C_18_H_32_O_5_ |
| 180 | 634.0812 | 19.61 | 6769984 | Corilagin | C_27_H_22_O_18_ |
| 181 | 146.0369 | 26.25 | 16769274 | Coumarin | C_9_H_6_O_2_ |
| 182 | 296.1413 | 40.37 | 23019477916 | Cryptotanshinone | C_19_H_20_O_3_ |
| 183 | 368.1261 | 34.83 | 443417139 | Curcumin | C_21_H_20_O_6_ |
| 184 | 520.3037 | 22.82 | 3071181755 | Cyasterone | C_29_H_44_O_8_ |
| 185 | 244.1213 | 21.98 | 16273776 | Cyclo(phenylalanyl-prolyl) | C_14_H_16_N_2_O_2_ |
| 186 | 180.0634 | 1.56 | 188188291 | D-(-)-Fructose | C_6_H_12_O_6_ |
| 187 | 182.0790 | 1.49 | 959501871 | D-(-)-Mannitol | C_6_H_14_O_6_ |
| 188 | 192.0635 | 18.13 | 51476370 | D-(-)-Quinic acid | C_7_H_12_O_6_ |
| 189 | 150.0527 | 1.72 | 56322006 | D-(-)-Ribose | C_5_H_10_O_5_ |
| 190 | 254.0580 | 24.85 | 35468658 | Daidzein | C_15_H_10_O_4_ |
| 191 | 198.0529 | 16.56 | 432222938 | Danshensu | C_9_H_10_O_5_ |
| 192 | 365.1630 | 25.44 | 12526599 | Dehydrocorydaline | C_22_H_23_NO_4_ |
| 193 | 270.1985 | 46.96 | 18739920 | Dehydroepiandrosterone (DHEA) | C_19_H_28_O_2_ |
| 194 | 454.3449 | 31.90 | 17768915 | Dehydrotrametenolic acid | C_30_H_46_O_3_ |
| 195 | 338.1156 | 34.28 | 324372357 | Demethoxycurcumin | C_20_H_18_O_5_ |
| 196 | 278.0943 | 36.76 | 2983274654 | Dihydrotanshinone I | C_18_H_14_O_3_ |
| 197 | 128.0587 | 1.85 | 67924352 | Dihydrothymine | C_5_H_8_N_2_O_2_ |
| 198 | 462.1166 | 20.36 | 7587122 | Diosmetin-7-O-β-D-glucopyranoside | C_22_H_22_O_11_ |
| 199 | 182.0580 | 17.90 | 29692084 | DL-4-Hydroxyphenyllactic acid | C_9_H_10_O_4_ |
| 200 | 174.1119 | 1.39 | 74917925 | DL-Arginine | C_6_H_14_N_4_O_2_ |
| 201 | 143.0948 | 1.82 | 183865270 | DL-Stachydrine | C_7_H_13_NO_2_ |
| 202 | 204.0902 | 17.87 | 42720427 | DL-Tryptophan | C_11_H_12_N_2_O_2_ |
| 203 | 328.2404 | 36.90 | 13555192 | Docosahexaenoic acid | C_22_H_32_O_2_ |
| 204 | 374.2822 | 37.50 | 53466346 | Docosahexaenoic acid ethyl ester | C_24_H_36_O_2_ |
| 205 | 342.2562 | 48.75 | 24124432 | Docosahexaenoic acid methyl ester | C_23_H_34_O_2_ |
| 206 | 230.1517 | 29.52 | 12632718 | Dodecanedioic acid | C_12_H_22_O_4_ |
| 207 | 266.1551 | 37.85 | 39620208 | Dodecyl sulfate | C_12_H_26_O_4_S |
| 208 | 284.2141 | 48.05 | 301324101 | Eicosapentaenoic acid | C_20_H_30_O_2_ |
| 209 | 178.0267 | 19.63 | 11777297 | Esculetin | C_9_H_6_O_4_ |
| 210 | 288.1726 | 33.33 | 17458613 | Estriol | C_18_H_24_O_3_ |
| 211 | 150.0683 | 19.63 | 14378072 | Ethyl benzoate | C_9_H_10_O_2_ |
| 212 | 222.0740 | 1.66 | 77804022 | Ethyl-β-D-glucuronide | C_8_H_14_O_7_ |
| 213 | 132.0422 | 3.59 | 16193745 | Ethylmalonic acid | C_5_H_8_O_4_ |
| 214 | 272.2142 | 48.18 | 4543567 | Etiocholanolone | C_19_H_30_O_2_ |
| 215 | 194.0581 | 22.33 | 495515852 | Ferulic acid | C_10_H_10_O_4_ |
| 216 | 268.0735 | 29.75 | 2005090567 | Formononetin | C_16_H_12_O_4_ |
| 217 | 170.0216 | 17.73 | 16335615 | Gallic acid | C_7_H_6_O_5_ |
| 218 | 270.0529 | 27.52 | 49961155 | Genistein | C_15_H_10_O_5_ |
| 219 | 356.1110 | 18.65 | 16790902 | Gentiopicrin | C_16_H_20_O_9_ |
| 220 | 154.0266 | 18.52 | 6801190 | Gentisic acid | C_7_H_6_O_4_ |
| 221 | 468.3244 | 45.82 | 152677073 | Glabrolide | C_30_H_44_O_4_ |
| 222 | 196.0582 | 1.53 | 157007633 | Gluconic acid | C_6_H_12_O_7_ |
| 223 | 130.0266 | 1.87 | 51507793 | Glutaconic acid | C_5_H_6_O_4_ |
| 224 | 453.2858 | 40.21 | 5194437 | Glycerophospho-N-palmitoyl ethanolamine | C_21_H_44_NO_7_P |
| 225 | 284.0685 | 33.29 | 21474355 | Glycitein | C_16_H_12_O_5_ |
| 226 | 1220.6187 | 31.36 | 39518674 | Hederacoside C | C_59_H_96_O_26_ |
| 227 | 286.2144 | 38.04 | 14516525 | Hexadecanedioic acid | C_16_H_30_O_4_ |
| 228 | 300.0634 | 29.28 | 68274259 | Hispidulin | C_16_H_12_O_6_ |
| 229 | 300.0634 | 27.91 | 33269485 | Hydroxygenkwanin | C_16_H_12_O_6_ |
| 230 | 205.0741 | 17.52 | 3221447 | Indole-3-lactic acid | C_11_H_11_NO_3_ |
| 231 | 357.0770 | 36.00 | 240898066 | Indomethacin | C_19_H_16_ClNO_4_ |
| 232 | 516.1270 | 22.97 | 199197820 | Isochlorogenic acid C | C_25_H_24_O_12_ |
| 233 | 194.0578 | 18.63 | 7959261 | Isoferulic acid | C_10_H_10_O_4_ |
| 234 | 464.1688 | 25.08 | 149225376 | Isomucronulatol 7-O-glucoside | C_23_H_28_O_10_ |
| 235 | 166.0265 | 18.73 | 50779278 | Isophthalic acid | C_8_H_6_O_4_ |
| 236 | 287.2825 | 32.23 | 136223764 | Isopropyl myristate | C_17_H_34_O_2_ |
| 237 | 129.0580 | 1.89 | 5364780 | Isoquinoline | C_9_H_7_N |
| 238 | 130.0266 | 1.73 | 20136620 | Itaconic acid | C_5_H_6_O_4_ |
| 239 | 286.0478 | 27.89 | 41509507 | Kaempferol | C_15_H_10_O_6_ |
| 240 | 448.1009 | 22.59 | 37545252 | Kaempferol-7-O-β-D-glucopyranoside | C_21_H_20_O_11_ |
| 241 | 161.0690 | 1.38 | 44858153 | Kanosamine | C_6_H_13_NO_5_ |
| 242 | 134.0215 | 1.85 | 396511598 | L-(-)-Malic acid | C_4_H_6_O_5_ |
| 243 | 133.0374 | 1.43 | 8139280 | L-Aspartic acid | C_4_H_7_NO_4_ |
| 244 | 119.0583 | 1.45 | 25069733 | L-Homoserine | C_4_H_9_NO_3_ |
| 245 | 148.0527 | 1.88 | 227150437 | L-Phenylalanine | C_9_H_11_NO_2_ |
| 246 | 129.0427 | 1.45 | 38051449 | L-Pyroglutamic acid | C_5_H_7_NO_3_ |
| 247 | 136.0371 | 1.54 | 235705269 | L-Threonic acid | C_4_H_8_O_5_ |
| 248 | 522.2103 | 21.47 | 16810487 | Lariciresinol 4-O-glucoside | C_26_H_34_O_11_ |
| 249 | 285.1014 | 28.26 | 79260932146 | Letrozole | C_17_H_11_N_5_ |
| 250 | 380.1990 | 43.73 | 253988272 | Levistilide A | C_24_H_28_O_4_ |
| 251 | 550.1692 | 21.47 | 3246737 | Liguiritigenin-7-O-β-D-apiosyl-4'-O-β-D-glucoside | C_26_H_30_O_13_ |
| 252 | 190.0995 | 36.84 | 837373681 | Ligustilide | C_12_H_14_O_2_ |
| 253 | 323.2827 | 44.30 | 152217608 | Linoleoyl Ethanolamide | C_20_H_37_NO_2_ |
| 254 | 256.0735 | 29.36 | 29752971 | Liquiritigenin | C_15_H_12_O_4_ |
| 255 | 538.1114 | 23.83 | 488050310 | Lithospermic acid | C_27_H_22_O_12_ |
| 256 | 376.1371 | 16.73 | 179321378 | Loganic acid | C_16_H_24_O_10_ |
| 257 | 436.1583 | 19.67 | 20819805 | Loganin | C_17_H_26_O_10_ |
| 258 | 266.1306 | 38.89 | 34612640 | Magnolol | C_18_H_18_O_2_ |
| 259 | 116.0109 | 1.85 | 69905895 | Maleic acid | C_4_H_4_O_4_ |
| 260 | 504.1692 | 1.82 | 109959922 | Manninotriose | C_18_H_32_O_16_ |
| 261 | 344.2352 | 35.08 | 36939284 | Medroxyprogesterone | C_22_H_32_O_3_ |
| 262 | 161.1255 | 48.54 | 101659617 | Medrysone | C_22_H_32_O_3_ |
| 263 | 304.2403 | 48.55 | 664653363 | Mesterolone | C_20_H_32_O_2_ |
| 264 | 278.1520 | 26.31 | 27013640 | methyl 2-[4-ethenyl-2,6-dihydroxy-3-(3-hydroxyprop-1-en-2-yl)-4-methylcyclohexyl]prop-2-enoate | C_16_H_24_O_5_ |
| 265 | 287.2825 | 32.86 | 101706928 | Methyl palmitate | C_17_H_34_O_2_ |
| 266 | 374.1003 | 25.02 | 158032247 | Methyl rosmarinate | C_19_H_18_O_8_ |
| 267 | 462.1529 | 24.72 | 40459097 | Methylnissolin-3-O-glucoside | C_23_H_26_O_10_ |
| 268 | 294.1866 | 44.18 | 16323240 | Myristyl sulfate | C_14_H_30_O_4_S |
| 269 | 246.1004 | 22.00 | 8125682 | N-Acetyl-DL-tryptophan | C_13_H_14_N_2_O_3_ |
| 270 | 207.0895 | 21.63 | 6353283 | N-Acetyl-L-phenylalanine | C_11_H_13_NO_3_ |
| 271 | 311.1158 | 22.23 | 10336953 | N-Feruloyloctopamine | C_18_H_19_NO_5_ |
| 272 | 624.1696 | 22.21 | 29844661 | Narcissoside | C_28_H_32_O_16_ |
| 273 | 272.0685 | 27.35 | 39486340 | Naringenin | C_15_H_12_O_5_ |
| 274 | 354.0952 | 17.82 | 56375320 | Neochlorogenic acid | C_16_H_18_O_9_ |
| 275 | 122.0480 | 1.85 | 96081348 | Nicotinamide | C_6_H_6_N_2_O |
| 276 | 302.2246 | 48.54 | 94766053 | Norethandrolone | C_20_H_30_O_2_ |
| 277 | 262.2299 | 46.07 | 127924056 | octadec-9-ynoic acid | C_18_H_32_O_2_ |
| 278 | 418.3083 | 51.79 | 244427666 | Octyl decyl phthalate | C_26_H_42_O_4_ |
| 279 | 281.2721 | 51.67 | 11245505 | Oleamide | C_18_H_35_NO |
| 280 | 454.3450 | 35.24 | 305782506 | Oleanonic acid | C_30_H_46_O_3_ |
| 281 | 325.2983 | 46.26 | 131861402 | Oleoyl ethanolamide | C_20_H_39_NO_2_ |
| 282 | 430.1267 | 23.94 | 304616893 | Ononin | C_22_H_22_O_9_ |
| 283 | 244.1102 | 37.30 | 16490910 | Osthol | C_15_H_16_O_3_ |
| 284 | 460.1584 | 17.97 | 11849818 | Paeonolide | C_20_H_28_O_12_ |
| 285 | 299.2825 | 45.55 | 64809282 | Palmitoyl ethanolamide | C_18_H_37_NO_2_ |
| 286 | 219.1108 | 1.97 | 18297221 | Pantothenic acid | C_9_H_17_NO_5_ |
| 287 | 248.1413 | 29.83 | 14499383 | Parthenolide | C_15_H_20_O_3_ |
| 288 | 134.0732 | 27.10 | 16791110 | Phenylacetone | C_9_H_10_O |
| 289 | 436.1370 | 23.33 | 11769167 | Phloridzin | C_21_H_24_O_10_ |
| 290 | 160.0735 | 19.37 | 8946037 | Pimelic acid | C_7_H_12_O_4_ |
| 291 | 520.1946 | 22.27 | 24745180 | Pinoresinol 4-O-glucoside | C_26_H_32_O_11_ |
| 292 | 594.1952 | 24.06 | 17167392 | Poncirin | C_28_H_34_O_14_ |
| 293 | 115.0634 | 1.69 | 1215564504 | Proline | C_5_H_9_NO_2_ |
| 294 | 336.2301 | 50.20 | 131957163 | Prostaglandin E1 | C_20_H_34_O_5_ |
| 295 | 336.2301 | 33.84 | 124802519 | Prostaglandin H1 | C_20_H_34_O_5_ |
| 296 | 332.1988 | 37.96 | 24906039 | Prostaglandin K2 | C_20_H_30_O_5_ |
| 297 | 138.0317 | 18.19 | 1399344319 | Protocatechualdehyde | C_7_H_6_O_3_ |
| 298 | 126.0318 | 1.83 | 117211102 | Pyrogallol | C_6_H_6_O_3_ |
| 299 | 464.0959 | 21.10 | 14946195 | Quercetin-3β-D-glucoside | C_21_H_20_O_12_ |
| 300 | 486.3349 | 32.95 | 21862261 | Quillaic acid | C_30_H_46_O_5_ |
| 301 | 550.1747 | 1.79 | 198677780 | Raffinose | C_18_H_32_O_16_ |
| 302 | 152.0474 | 16.53 | 5911844 | Resorcinol monoacetate | C_8_H_8_O_3_ |
| 303 | 376.1385 | 19.15 | 12939854 | Riboflavin | C_17_H_20_N_4_O_6_ |
| 304 | 360.0845 | 23.11 | 327660372 | Rosmarinic acid | C_18_H_16_O_8_ |
| 305 | 610.1538 | 21.04 | 19612523 | Rutin | C_27_H_30_O_16_ |
| 306 | 612.1696 | 17.78 | 13885089 | Safflomin A | C_27_H_32_O_16_ |
| 307 | 179.0946 | 1.85 | 271453594 | Salsolinol | C_10_H_13_NO_2_ |
| 308 | 494.1213 | 24.71 | 6848441873 | Salvianolic acid A | C_26_H_22_O_10_ |
| 309 | 735.1800 | 22.81 | 34886622 | Salvianolic acid B | C_36_H_30_O_16_ |
| 310 | 492.1058 | 26.76 | 150015434 | Salvianolic acid C | C_26_H_20_O_10_ |
| 311 | 206.0581 | 24.25 | 338873719 | Scoparone | C_11_H_10_O_4_ |
| 312 | 314.0790 | 31.83 | 110147994 | Scrophulein | C_17_H_14_O_6_ |
| 313 | 194.1308 | 36.57 | 83085806 | Sedanolide | C_12_H_18_O_2_ |
| 314 | 192.1152 | 33.98 | 1091706330 | Senkyunolide A | C_12_H_16_O_2_ |
| 315 | 206.0944 | 24.40 | 269601130 | Senkyunolide H | C_12_H_16_O_4_ |
| 316 | 276.1728 | 33.95 | 13825027 | Shogaol | C_17_H_24_O_3_ |
| 317 | 942.5190 | 31.17 | 110362320 | Soyasaponin I | C_48_H_78_O_18_ |
| 318 | 666.2222 | 1.84 | 224997151 | Stachyose | C_24_H_42_O_21_ |
| 319 | 174.0891 | 21.34 | 32962101 | Suberic acid | C_8_H_14_O_4_ |
| 320 | 118.0265 | 1.85 | 356053082 | Succinic acid | C_4_H_6_O_4_ |
| 321 | 342.1161 | 1.79 | 4506216033 | Sucrose | C_12_H_22_O_11_ |
| 322 | 404.1320 | 19.24 | 233436242 | Sweroside | C_16_H_22_O_9_ |
| 323 | 294.1257 | 44.13 | 24762015621 | Tanshinone IIA | C_19_H_18_O_3_ |
| 324 | 462.1165 | 19.78 | 4802037 | Tectoridin | C_22_H_22_O_11_ |
| 325 | 300.0634 | 28.34 | 87542357 | Tectorigenin | C_16_H_12_O_6_ |
| 326 | 166.0265 | 19.16 | 82688351 | Terephthalic acid | C_8_H_6_O_4_ |
| 327 | 258.1832 | 33.82 | 5047312 | Tetradecanedioic acid | C_14_H_26_O_4_ |
| 328 | 136.1002 | 18.56 | 22281919 | Tetramethylpyrazine | C_8_H_12_N_2_ |
| 329 | 187.0636 | 17.81 | 63841631 | trans-3-Indoleacrylic acid | C_11_H_9_NO_2_ |
| 330 | 248.1804 | 48.13 | 3642855 | Trenbolone | C_18_H_22_O_2_ |
| 331 | 448.1006 | 22.43 | 6423260 | Trifolin | C_21_H_20_O_11_ |
| 332 | 137.0479 | 1.76 | 286743745 | Trigonelline | C_7_H_7_NO_2_ |
| 333 | 498.3711 | 50.41 | 48272542 | Tsugaric acid A | C_32_H_50_O_4_ |
| 334 | 244.0694 | 1.86 | 49107927 | Uridine | C_9_H_12_N_2_O_6_ |
| 335 | 454.3451 | 41.98 | 74275920 | Ursonic acid | C_30_H_46_O_3_ |
| 336 | 152.0473 | 19.40 | 10520941 | Vanillin | C_8_H_8_O_3_ |
| 337 | 578.1642 | 20.73 | 32493101 | Vitexin rhamnoside | C_27_H_30_O_14_ |
| 338 | 432.1056 | 21.46 | 9509515 | Vitexin | C_21_H_20_O_10_ |
| 339 | 314.0428 | 29.99 | 19139617 | Wedelolactone | C_16_H_10_O_7_ |
| 340 | 454.3449 | 34.12 | 75053068 | Wilforlide A | C_30_H_46_O_3_ |
| 341 | 388.1736 | 19.68 | 27849757 | {(1R,2R)-2-[(2Z)-5-(Hexopyranosyloxy)-2-penten-1-yl]-3-oxocyclopentyl}acetic acid | C_18_H_28_O_9_ |
| 342 | 359.1430 | 1.77 | 57735239 | α-Lactose | C_12_H_22_O_11_ |
| 343 | 321.2669 | 41.98 | 22073169 | α-Linolenoyl ethanolamide | C_20_H_35_NO_2_ |
| 344 | 178.0477 | 1.73 | 97486381 | δ-Gluconic acid δ-lactone | C_6_H_10_O_6_ |

**Supplementary Table 4.** UPLC-MS/MS identification results of the components of YXB that can be absorbed by the intestinal.

| No. | detected Mass (Da) | tR (min) | Area (Average) | Identification | Molecular formula |
| --- | --- | --- | --- | --- | --- |
| 1 | 129.0426 | 3.68 | 26887879610 | 4-Oxoproline | C_5_H_7_NO_3_ |
| 2 | 198.0528 | 16.30 | 23438828930 | Danshensu | C_9_H_10_O_5_ |
| 3 | 138.0316 | 18.06 | 22111692247 | Protocatechualdehyde | C_7_H_6_O_3_ |
| 4 | 330.2406 | 28.47 | 21189668878 | (15Z)-9,12,13-Trihydroxy-15-octadecenoic acid | C_18_H_34_O_5_ |
| 5 | 206.0943 | 23.88 | 17668037185 | Senkyunolide H | C_12_H_16_O_4_ |
| 6 | 117.0790 | 1.56 | 14837174292 | Betaine | C_5_H_11_NO_2_ |
| 7 | 180.0422 | 23.21 | 13490503308 | Caffeic acid | C_9_H_8_O_4_ |
| 8 | 187.0634 | 17.63 | 10570802185 | trans-3-Indoleacrylic acid | C_11_H_9_NO_2_ |
| 9 | 494.1213 | 23.31 | 9887521074 | Salvianolic acid A | C_26_H_22_O_10_ |
| 10 | 520.3033 | 22.89 | 8973966304 | Cyasterone | C_29_H_44_O_8_ |
| 11 | 103.0997 | 1.71 | 8266415279 | Choline | C_5_H_13_NO |
| 12 | 190.0994 | 29.72 | 7997428129 | Ligustilide | C_12_H_14_O_2_ |
| 13 | 284.0684 | 25.63 | 7484034942 | Calycosin | C_16_H_12_O_5_ |
| 14 | 612.1692 | 18.81 | 5194628926 | Safflomin A | C_27_H_32_O_16_ |
| 15 | 354.0951 | 19.03 | 4913223767 | Chlorogenic acid | C_16_H_18_O_9_ |
| 16 | 166.0265 | 19.03 | 4623519552 | Terephthalic acid | C_8_H_6_O_4_ |
| 17 | 666.2219 | 2.36 | 4407180293 | Stachyose | C_24_H_42_O_21_ |
| 18 | 115.0634 | 1.82 | 4369754154 | Proline | C_5_H_9_NO_2_ |
| 19 | 192.1152 | 33.94 | 4003620966 | Senkyunolide A | C_12_H_16_O_2_ |
| 20 | 248.1413 | 30.66 | 3930680504 | Parthenolide | C_15_H_20_O_3_ |
| 21 | 109.0528 | 3.27 | 3879558315 | 3-Hydroxy-2-methylpyridine | C_6_H_7_NO |
| 22 | 326.1915 | 44.69 | 3822103379 | 4-Dodecylbenzenesulfonic acid | C_18_H_30_O_3_S |
| 23 | 328.2248 | 27.28 | 3627345503 | Corchorifatty acid F | C_18_H_32_O_5_ |
| 24 | 538.1110 | 23.30 | 3623600234 | Lithospermic acid | C_27_H_22_O_12_ |
| 25 | 354.0951 | 17.71 | 3339750055 | Neochlorogenic acid | C_16_H_18_O_9_ |
| 26 | 124.0524 | 19.30 | 3017111569 | 2-Hydroxybenzyl alcohol | C_7_H_8_O_2_ |
| 27 | 126.0318 | 16.78 | 2824900088 | Pyrogallol | C_6_H_6_O_3_ |
| 28 | 134.0215 | 2.17 | 2783214229 | L-(-)-Malic acid | C_4_H_6_O_5_ |
| 29 | 504.1690 | 2.51 | 2447787500 | D-Raffinose | C_18_H_32_O_16_ |
| 30 | 136.0524 | 16.30 | 2403826721 | 2-Methylbenzoic acid | C_8_H_8_O_2_ |
| 31 | 220.1828 | 30.05 | 2344922907 | (-)-Caryophyllene oxide | C_15_H_24_O |
| 32 | 250.1569 | 34.25 | 2339750769 | 2-[(2S,4aR,8aS)-2-Hydroxy-4a-methyl-8-methylenedecahydro-2-naphthalenyl]acrylic acid | C_15_H_22_O_3_ |
| 33 | 830.4666 | 29.13 | 2286661237 | Astragaloside IV | C_41_H_68_O_14_ |
| 34 | 222.0891 | 26.87 | 2283493516 | 4-(4-Ethoxyphenyl)-4-oxobutanoic acid | C_12_H_14_O_4_ |
| 35 | 446.1214 | 21.45 | 2251246924 | Calycosin-7-O-β-D-glucoside | C_22_H_22_O_10_ |
| 36 | 296.1410 | 40.35 | 2105213976 | Cryptotanshinone | C_19_H_20_O_3_ |
| 37 | 134.0367 | 29.74 | 1977774582 | Formononetin | C_16_H_12_O_4_ |
| 38 | 164.0474 | 21.36 | 1892078630 | 4-Coumaric acid | C_9_H_8_O_3_ |
| 39 | 152.0474 | 21.20 | 1767619118 | Vanillin | C_8_H_8_O_3_ |
| 40 | 492.1058 | 26.80 | 1741362078 | Salvianolic acid C | C_26_H_20_O_10_ |
| 41 | 194.0579 | 24.98 | 1444198749 | Isoferulic acid | C_10_H_10_O_4_ |
| 42 | 122.0365 | 26.18 | 1393646421 | Benzoic acid | C_7_H_6_O_2_ |
| 43 | 138.0316 | 23.52 | 1371697488 | 3-Hydroxybenzoic acid | C_7_H_6_O_3_ |
| 44 | 306.1102 | 22.03 | 1329538694 | Cimifugin | C_16_H_18_O_6_ |
| 45 | 356.2565 | 30.37 | 1328157231 | 13,14-Dihydro prostaglandin E1 | C_20_H_36_O_5_ |
| 46 | 174.1117 | 1.80 | 1308430958 | DL-Arginine | C_6_H_14_N_4_O_2_ |
| 47 | 516.1268 | 20.20 | 1297542056 | 1,3-Dicaffeoylquinic acid | C_25_H_24_O_12_ |
| 48 | 430.1265 | 23.98 | 1260167849 | Ononin | C_22_H_22_O_9_ |
| 49 | 246.1003 | 21.97 | 1128793240 | N-Acetyl-DL-tryptophan | C_13_H_14_N_2_O_3_ |
| 50 | 192.0634 | 18.99 | 1094157989 | D-(-)-Quinic acid | C_7_H_12_O_6_ |
| 51 | 254.1518 | 30.31 | 853873960 | (+/-)-C75 | C_14_H_22_O_4_ |
| 52 | 154.0266 | 16.20 | 819095772 | 2,3-Dihydroxybenzoic acid | C_7_H_6_O_4_ |
| 53 | 202.1205 | 25.19 | 816201107 | 3-tert-Butyladipic acid | C_10_H_18_O_4_ |
| 54 | 278.0944 | 36.75 | 796062723 | Dihydrotanshinone I | C_18_H_14_O_3_ |
| 55 | 134.0732 | 26.76 | 759164220 | 2,4-Dimethylbenzaldehyde | C_9_H_10_O |
| 56 | 942.5190 | 31.26 | 704446865 | Soyasaponin I | C_48_H_78_O_18_ |
| 57 | 266.1518 | 31.66 | 702569694 | 1,9b-Dihydroxy-6,6,9a-trimethyl-5,5a,6,7,8,9,9a,9b-octahydronaphtho[1,2-c]furan-3(1H)-one | C_15_H_22_O_4_ |
| 58 | 454.3447 | 30.88 | 699116132 | Wilforlide A | C_30_H_46_O_3_ |
| 59 | 322.2510 | 40.95 | 687256057 | 11-Deoxy prostaglandin F1β | C_20_H_36_O_4_ |
| 60 | 168.0422 | 18.73 | 646932003 | 3-Methoxysalicylic acid | C_8_H_8_O_4_ |
| 61 | 480.1655 | 22.15 | 632374603 | Kaempferol-3-O-rutinoside | C_27_H_30_O_15_ |
| 62 | 284.0684 | 22.79 | 627996662 | Glycitein | C_16_H_12_O_5_ |
| 63 | 196.0582 | 1.78 | 585212881 | Gluconic acid | C_6_H_12_O_7_ |
| 64 | 194.1308 | 28.49 | 510198355 | Sedanolide | C_12_H_18_O_2_ |
| 65 | 137.0478 | 1.78 | 494818062 | Trigonelline | C_7_H_7_NO_2_ |
| 66 | 252.1725 | 33.25 | 491086610 | 2-(8-Hydroxy-4a,8-dimethyldecahydro-2-naphthalenyl)acrylic acid | C_15_H_24_O_3_ |
| 67 | 307.2147 | 25.51 | 467050860 | 10-Nitrolinoleate | C_18_H_31_NO_4_ |
| 68 | 230.1516 | 29.51 | 461560926 | Dodecanedioic acid | C_12_H_22_O_4_ |
| 69 | 207.0894 | 21.12 | 453700774 | N-Acetyl-L-phenylalanine | C_11_H_13_NO_3_ |
| 70 | 388.1734 | 19.69 | 453606527 | {(1R,2R)-2-[(2Z)-5-(Hexopyranosyloxy)-2-penten-1-yl]-3-oxocyclopentyl}acetic acid | C_18_H_28_O_9_ |
| 71 | 352.2249 | 30.63 | 444033058 | 15-keto Prostaglandin E1 | C_20_H_32_O_5_ |
| 72 | 254.0578 | 24.87 | 443599353 | Daidzein | C_15_H_10_O_4_ |
| 73 | 174.0891 | 21.33 | 440799766 | Suberic acid | C_8_H_14_O_4_ |
| 74 | 284.2142 | 33.24 | 388444911 | Eicosapentaenoic acid | C_20_H_30_O_2_ |
| 75 | 304.2403 | 36.46 | 387625470 | Mesterolone | C_20_H_32_O_2_ |
| 76 | 182.0580 | 17.77 | 382882835 | DL-4-Hydroxyphenyllactic acid | C_9_H_10_O_4_ |
| 77 | 578.1642 | 21.19 | 355377852 | Vitexin rhamnoside | C_27_H_30_O_14_ |
| 78 | 122.1096 | 30.57 | 336521062 | 1,2,3,4-Tetramethyl-1,3-cyclopentadiene | C_9_H_14_ |
| 79 | 314.2455 | 34.82 | 316745490 | (+/-)12(13)-DiHOME | C_18_H_34_O_4_ |
| 80 | 165.0789 | 16.01 | 314487364 | L-Phenylalanine | C_9_H_11_NO_2_ |
| 81 | 124.0524 | 16.29 | 310884637 | 3-Hydroxybenzyl alcohol | C_7_H_8_O_2_ |
| 82 | 294.1257 | 44.11 | 279399987 | Tanshinone IIA | C_19_H_18_O_3_ |
| 83 | 272.2352 | 45.94 | 269513083 | 16-Hydroxyhexadecanoic acid | C_16_H_32_O_3_ |
| 84 | 182.0580 | 21.69 | 257991967 | 3,5-Dimethoxy-4-hydroxybenzaldehyde | C_9_H_10_O_4_ |
| 85 | 464.1685 | 25.10 | 241391080 | Isomucronulatol 7-O-glucoside | C_23_H_28_O_10_ |
| 86 | 610.1536 | 21.41 | 230496920 | Rutin | C_27_H_30_O_16_ |
| 87 | 462.2468 | 21.23 | 229005340 | 2-(4-Methyl-3-cyclohexen-1-yl)-2-propanyl 6-O-(6-deoxy-α-L-mannopyranosyl)-β-D-glucopyranoside | C_22_H_38_O_10_ |
| 88 | 178.0477 | 3.70 | 224578543 | δ-Gluconic acid δ-lactone | C_6_H_10_O_6_ |
| 89 | 152.0473 | 16.27 | 223254436 | 4-Hydroxyphenylacetic acid | C_8_H_8_O_3_ |
| 90 | 284.2142 | 28.49 | 215892708 | (9cis)-Retinal | C_20_H_28_O |
| 91 | 164.0473 | 26.19 | 208394875 | 2-Hydroxycinnamic acid | C_9_H_8_O_3_ |
| 92 | 454.3447 | 31.93 | 201944803 | Dehydrotrametenolic acid | C_30_H_46_O_3_ |
| 93 | 264.2454 | 47.51 | 182289208 | Oleamide | C_18_H_35_NO |
| 94 | 109.0528 | 20.09 | 178299364 | 4-Aminophenol | C_6_H_7_NO |
| 95 | 286.1570 | 42.69 | 172064445 | 16α-Hydroxyestrone | C_18_H_22_O_3_ |
| 96 | 328.0795 | 18.40 | 155535318 | Bengenin | C_14_H_16_O_9_ |
| 97 | 161.0689 | 1.50 | 147553473 | Kanosamine | C_6_H_13_NO_5_ |
| 98 | 160.0735 | 19.34 | 142387663 | Pimelic acid | C_7_H_12_O_4_ |
| 99 | 126.0681 | 20.42 | 142226287 | 1-(Carboxymethyl)cyclohexanecarboxylic acid | C_9_H_14_O_4_ |
| 100 | 512.3508 | 48.01 | 141803043 | Acetyl-11-keto-β-boswellic acid | C_32_H_48_O_5_ |
| 101 | 358.1265 | 19.75 | 141736001 | 3-[2-(β-D-Glucopyranosyloxy)-4-methoxyphenyl]propanoic acid | C_16_H_22_O_9_ |
| 102 | 522.2104 | 21.50 | 130149460 | Lariciresinol 4-O-glucoside | C_26_H_34_O_11_ |
| 103 | 154.0266 | 18.37 | 124313980 | Gentisic acid | C_7_H_6_O_4_ |
| 104 | 146.0368 | 22.38 | 113939218 | Coumarin | C_9_H_6_O_2_ |
| 105 | 276.1726 | 32.45 | 108318170 | Shogaol | C_17_H_24_O_3_ |
| 106 | 464.0958 | 21.84 | 106026805 | Quercetin-3β-D-glucoside | C_21_H_20_O_12_ |
| 107 | 216.0898 | 18.93 | 99596456 | 2,3,4,9-Tetrahydro-1H-β-carboline-3-carboxylic acid | C_12_H_12_N_2_O_2_ |
| 108 | 460.1581 | 18.89 | 97839151 | Paeonolide | C_20_H_28_O_12_ |
| 109 | 182.0580 | 19.08 | 93634536 | 3,4-Dihydroxyphenylpropionic acid | C_9_H_10_O_4_ |
| 110 | 152.0473 | 19.38 | 91079872 | Resorcinol monoacetate | C_8_H_8_O_3_ |
| 111 | 374.1577 | 18.74 | 73905999 | 4-(3-Hydroxybutyl)phenyl β-D-glucopyranoside | C_16_H_24_O_7_ |
| 112 | 131.0736 | 19.00 | 45050112 | 3-Phenylpropionitrile | C_9_H_9_N |
| 113 | 346.2510 | 42.00 | 39024903 | Ciprostene | C_22_H_36_O_4_ |
| 114 | 344.2353 | 44.15 | 19125739 | Medrysone | C_22_H_32_O_3_ |
| 115 | 346.2510 | 45.76 | 17945176 | 16,16-Dimethyl prostaglandin A1 | C_22_H_36_O_4_ |
| 116 | 380.1988 | 39.61 | 14243223 | Levistilide A | C_24_H_28_O_4_ |

**Supplementary Table 5.** Western medical disease that YXB are used to treat and quantity of western medicines for disease treatment.

| Disease name | Number of mentions in the survey | Total number of therapeutic Western medicines |
| --- | --- | --- |
| osteoarthritis | 34 | 48 |
| lumbar disc herniation | 30 | 42 |
| swelling and pain after fracture | 28 | 44 |
| cervical spondylosis | 26 | 55 |
| frozen shoulder | 20 | 33 |
| tenosynovitis | 11 | 33 |
| Cardiovascular and cerebrovascular diseases such as stroke, cerebral infarction, etc. | 10 | 58^*^ |
| gout | 6 | 48 |
| rheumatoid arthritis | 6 | 61 |
| ankylosing spondylitis | 5 | 38 |
| Osteoporosis | 3 | 34 |
| Reactive arthritis | 3 | 39 |
| Avascular Necrosis of Femoral Head | 2 | 21 |
| spinal stenosis | 1 | 8 |
| scleroderma | 1 | 65 |
| Psoriatic arthritis | 1 | 46 |
| Fibromyalgia | 1 | 37 |
| Systemic lupus erythematosus | 1 | 52 |
| Dysmenorrhea | 1 | 36 |
| Diabetic peripheral neuropathy | 1 | 27 |
| Sjogren's syndrome | 1 | 30 |
| Vasculitis or systemic vasculitis^#^ | 1 | 22 |

Note: ^*^ Only western medicines for the treatment of stroke, sequelae of cerebral infarction and stroke prevention were included. ^#^ YXB may have a therapeutic effect on this disease.

**Supplementary Table 6.** YXB’s clinical indications and corresponding treatment western drugs

| Disease name | drug | No. of drugs |
| --- | --- | --- |
| osteoarthritis | piroxicam | 1 |
|  | etodolac | 2 |
|  | meloxicam | 3 |
|  | nabumetone | 4 |
|  | tenoxicam | 5 |
|  | gabapentin | 6 |
|  | celecoxib | 7 |
|  | adalimumab | 8 |
|  | etoricoxib | 9 |
|  | lumiracoxib | 10 |
|  | duloxetine | 11 |
|  | pelubiprofen | 12 |
|  | ademetionine | 13 |
|  | polmacoxib | 14 |
|  | strontium ranelate | 15 |
|  | aceclofenac | 16 |
|  | eptotermin alfa | 17 |
|  | tonogenchoncel-l | 18 |
|  | hyaluronate sodium | 19 |
|  | Cartistem | 20 |
|  | once-daily naproxen | 21 |
|  | Sinbaro | 22 |
|  | esflurbiprofen | 23 |
|  | paracetamol | 24 |
|  | tramadol | 25 |
|  | colchicine | 26 |
|  | fish oil ; | 27 |
|  | ergocalciferol | 28 |
|  | colecalciferol | 29 |
|  | glucosamine | 30 |
|  | chondroitin sulfate sodium | 31 |
|  | hydroxychloroquine | 32 |
|  | methotrexate | 33 |
|  | botulinum toxin | 34 |
|  | mecobalamin | 35 |
|  | cyanocobalamin | 36 |
|  | rofecoxib | 37 |
|  | nimesulide | 38 |
|  | acemetacin | 39 |
|  | diclofenac potassium | 40 |
|  | flurbiprofen | 41 |
|  | parecoxib | 42 |
|  | ibuprofen | 43 |
|  | indometacin | 44 |
|  | mefenamic acid | 45 |
|  | dexibuprofen | 46 |
|  | imidazole salicylate | 47 |
|  | ketoprofen | 48 |
| lumbar disc herniation | adalimumab | 1 |
|  | duloxetine | 2 |
|  | condoliase | 3 |
|  | paracetamol | 4 |
|  | ibuprofen | 5 |
|  | celecoxib | 6 |
|  | etoricoxib | 7 |
|  | gabapentin | 8 |
|  | pregabalin | 9 |
|  | tramadol | 10 |
|  | oxycodone hydrochloride | 11 |
|  | fentanyl | 12 |
|  | buprenorphine | 13 |
|  | mannitol | 14 |
|  | methylprednisolone | 15 |
|  | cortisone | 16 |
|  | hydrocortisone | 17 |
|  | betamethasone | 18 |
|  | dexamethasone | 19 |
|  | prednisone | 20 |
|  | prednisolone | 21 |
|  | eperisone | 22 |
|  | chlorzoxazone | 23 |
|  | mecobalamin | 24 |
|  | thiamine | 25 |
|  | cyanocobalamin | 26 |
|  | clopidogrel | 27 |
|  | aspirin | 28 |
|  | triamcinolone | 29 |
|  | pethidine | 30 |
|  | meloxicam | 31 |
|  | piroxicam | 32 |
|  | nabumetone | 33 |
|  | nimesulide | 34 |
|  | aceclofenac | 35 |
|  | diclofenac potassium | 36 |
|  | flurbiprofen | 37 |
|  | parecoxib | 38 |
|  | indometacin | 39 |
|  | mefenamic acid | 40 |
|  | dexibuprofen | 41 |
|  | ketoprofen | 42 |
| swelling and pain after fracture | alprostadil | 1 |
|  | nimodipine | 2 |
|  | nitroprusside sodium | 3 |
|  | sodium aescinate | 4 |
|  | nadroparin calcium | 5 |
|  | dalteparin sodium | 6 |
|  | enoxaparin sodium | 7 |
|  | diosmin | 8 |
|  | imrecoxib | 9 |
|  | clopidogrel | 10 |
|  | teriparatide | 11 |
|  | mannitol | 12 |
|  | dexamethasone | 13 |
|  | diclofenac sodium | 14 |
|  | parecoxib | 15 |
|  | hydromorphone hydrochloride | 16 |
|  | fentanyl | 17 |
|  | tramadol | 18 |
|  | pethidine | 19 |
|  | ketamine | 20 |
|  | bupivacaine | 21 |
|  | aspirin | 22 |
|  | indometacin | 23 |
|  | naproxen | 24 |
|  | celecoxib | 25 |
|  | nimesulide | 26 |
|  | piroxicam | 27 |
|  | meloxicam | 28 |
|  | procainamide | 29 |
|  | lidocaine | 30 |
|  | oxycodone hydrochloride | 31 |
|  | buprenorphine | 32 |
|  | paracetamol | 33 |
|  | nabumetone | 34 |
|  | rofecoxib | 35 |
|  | etoricoxib | 36 |
|  | aceclofenac | 37 |
|  | diclofenac potassium | 38 |
|  | flurbiprofen | 39 |
|  | ibuprofen | 40 |
|  | mefenamic acid | 41 |
|  | etodolac | 42 |
|  | dexibuprofen | 43 |
|  | ketoprofen | 44 |
| cervical spondylosis | celecoxib | 1 |
|  | methylprednisolone | 2 |
|  | cortisone | 3 |
|  | hydrocortisone | 4 |
|  | betamethasone | 5 |
|  | dexamethasone | 6 |
|  | prednisone | 7 |
|  | prednisolone | 8 |
|  | triamcinolone acetonide | 9 |
|  | triamcinolone | 10 |
|  | duloxetine | 11 |
|  | pregabalin | 12 |
|  | limaprost | 13 |
|  | aspirin | 14 |
|  | indometacin | 15 |
|  | sulindac | 16 |
|  | naproxen | 17 |
|  | ibuprofen | 18 |
|  | diclofenac sodium | 19 |
|  | paracetamol | 20 |
|  | rofecoxib | 21 |
|  | mefenamic acid | 22 |
|  | piroxicam | 23 |
|  | meloxicam | 24 |
|  | nabumetone | 25 |
|  | nimesulide | 26 |
|  | eperisone | 27 |
|  | chlorzoxazone | 28 |
|  | aspirin | 29 |
|  | ibuprofen | 30 |
|  | acemetacin | 31 |
|  | phenprobamate | 32 |
|  | benzydamine hydrochloride | 33 |
|  | kallidinogenase | 34 |
|  | bendazol | 35 |
|  | trihexyphenidyl | 36 |
|  | fosphenytoin sodium | 37 |
|  | oryzanol | 38 |
|  | nabumetone | 39 |
|  | chlorzoxazone | 40 |
|  | diazepam | 41 |
|  | mannitol | 42 |
|  | furosemide | 43 |
|  | mecobalamin | 44 |
|  | cobamamide | 45 |
|  | cyanocobalamin | 46 |
|  | etoricoxib | 47 |
|  | aceclofenac | 48 |
|  | diclofenac potassium | 49 |
|  | flurbiprofen | 50 |
|  | parecoxib | 51 |
|  | etodolac | 52 |
|  | dexibuprofen | 53 |
|  | ketoprofen | 54 |
|  | alprostadil | 55 |
| frozen shoulder | celecoxib | 1 |
|  | hyaluronate sodium | 2 |
|  | aspirin | 3 |
|  | indometacin | 4 |
|  | sulindac | 5 |
|  | naproxen | 6 |
|  | ibuprofen | 7 |
|  | diclofenac sodium | 8 |
|  | paracetamol | 9 |
|  | rofecoxib | 10 |
|  | mefenamic acid | 11 |
|  | piroxicam | 12 |
|  | meloxicam | 13 |
|  | nabumetone | 14 |
|  | nimesulide | 15 |
|  | cortisone acetate | 16 |
|  | cortisone | 17 |
|  | hydrocortisone | 18 |
|  | betamethasone | 19 |
|  | dexamethasone | 20 |
|  | methylprednisolone | 21 |
|  | prednisolone | 22 |
|  | prednisone | 23 |
|  | triamcinolone | 24 |
|  | etoricoxib | 25 |
|  | aceclofenac | 26 |
|  | acemetacin | 27 |
|  | diclofenac potassium | 28 |
|  | flurbiprofen | 29 |
|  | parecoxib | 30 |
|  | etodolac | 31 |
|  | dexibuprofen | 32 |
|  | ketoprofen | 33 |
| tenosynovitis | celecoxib | 1 |
|  | aspirin | 2 |
|  | indometacin | 3 |
|  | sulindac | 4 |
|  | naproxen | 5 |
|  | ibuprofen | 6 |
|  | diclofenac sodium | 7 |
|  | paracetamol | 8 |
|  | rofecoxib | 9 |
|  | mefenamic acid | 10 |
|  | piroxicam | 11 |
|  | meloxicam | 12 |
|  | nabumetone | 13 |
|  | nimesulide | 14 |
|  | botulinum toxin | 15 |
|  | triamcinolone acetonide | 16 |
|  | triamcinolone | 17 |
|  | betamethasone | 18 |
|  | cortisone | 19 |
|  | hydrocortisone | 20 |
|  | methylprednisolone | 21 |
|  | prednisone | 22 |
|  | prednisolone | 23 |
|  | hyaluronate sodium | 24 |
|  | dexamethasone | 25 |
|  | etoricoxib | 26 |
|  | aceclofenac | 27 |
|  | acemetacin | 28 |
|  | diclofenac potassium | 29 |
|  | flurbiprofen | 30 |
|  | etodolac | 31 |
|  | dexibuprofen | 32 |
|  | ketoprofen | 33 |
| Stroke | urokinase | 1 |
|  | butylphthalide | 2 |
|  | mannitol | 3 |
|  | sodium etacrynate | 4 |
|  | fructose diphosphate sodium | 5 |
|  | citicoline | 6 |
|  | alteplase | 7 |
|  | nimodipine | 8 |
|  | aniracetam | 9 |
|  | nilvadipine | 10 |
|  | cilostazol | 11 |
|  | argatroban | 12 |
|  | masitinib | 13 |
|  | citicoline | 14 |
|  | atorvastatin | 15 |
|  | rosuvastatin | 16 |
|  | prasugrel | 17 |
|  | TBN | 18 |
|  | prourokinase | 19 |
|  | NAL-8010 | 20 |
|  | Poly ICLC | 21 |
|  | tenecteplase | 22 |
|  | Cerebrolysin | 23 |
|  | STR-01 | 24 |
|  | clopidogrel | 25 |
|  | alteplase | 26 |
| Stroke preventive | nisoldipine | 27 |
|  | benidipine | 28 |
|  | lacidipine | 29 |
|  | lidoflazine | 30 |
|  | flunarizine | 31 |
|  | verapamil | 32 |
|  | diltiazem | 33 |
|  | nimodipine | 34 |
|  | lercanidipine | 35 |
|  | amlodipine | 36 |
|  | nifedipine | 37 |
|  | losartan | 38 |
|  | telmisartan | 39 |
|  | pravastatin sodium | 40 |
|  | lovastatin | 41 |
|  | simvastatin | 42 |
|  | fluvastatin | 43 |
|  | warfarin | 44 |
|  | dabigatran etexilate | 45 |
|  | rivaroxaban | 46 |
|  | apixaban | 47 |
|  | ezetimibe | 48 |
|  | nicotinic acid | 49 |
|  | fenofibrate | 50 |
|  | ciprofibrate | 51 |
|  | bezafibrate | 52 |
| sequelae of cerebral infarction | dabigatran etexilate | 53 |
|  | edaravone | 54 |
|  | aspirin | 55 |
|  | vinpocetine | 56 |
|  | oxiracetam | 57 |
|  | nimodipine | 58 |
| gout | pegloticase | 1 |
|  | etoricoxib | 2 |
|  | febuxostat | 3 |
|  | anakinra | 4 |
|  | canakinumab | 5 |
|  | topiroxostat | 6 |
|  | lesinurad | 7 |
|  | dotinurad | 8 |
|  | once-daily naproxen | 9 |
|  | colchicine | 10 |
|  | aspirin | 11 |
|  | indometacin | 12 |
|  | sulindac | 13 |
|  | naproxen | 14 |
|  | ibuprofen | 15 |
|  | diclofenac sodium | 16 |
|  | paracetamol | 17 |
|  | rofecoxib | 18 |
|  | mefenamic acid | 19 |
|  | piroxicam | 20 |
|  | meloxicam | 21 |
|  | nabumetone | 22 |
|  | nimesulide | 23 |
|  | celecoxib | 24 |
|  | methylprednisolone | 25 |
|  | cortisone | 26 |
|  | hydrocortisone | 27 |
|  | betamethasone | 28 |
|  | dexamethasone | 29 |
|  | prednisone | 30 |
|  | prednisolone | 31 |
|  | triamcinolone acetonide | 32 |
|  | triamcinolone | 33 |
|  | allopurinol | 34 |
|  | benzbromarone | 35 |
|  | infliximab | 36 |
|  | etanercept | 37 |
|  | sodium citrate | 38 |
|  | potassium citrate | 39 |
|  | sodium bicarbonate | 40 |
|  | aceclofenac | 41 |
|  | acemetacin | 42 |
|  | diclofenac potassium | 43 |
|  | flurbiprofen | 44 |
|  | parecoxib | 45 |
|  | etodolac | 46 |
|  | dexibuprofen | 47 |
|  | ketoprofen | 48 |
| rheumatoid arthritis | piroxicam | 1 |
|  | etodolac | 2 |
|  | leflunomide | 3 |
|  | meloxicam | 4 |
|  | nabumetone | 5 |
|  | tacrolimus | 6 |
|  | deflazacort | 7 |
|  | tenoxicam | 8 |
|  | rofecoxib | 9 |
|  | celecoxib | 10 |
|  | etanercept | 11 |
|  | adalimumab | 12 |
|  | etoricoxib | 13 |
|  | lumiracoxib | 14 |
|  | anakinra | 15 |
|  | pelubiprofen | 16 |
|  | infliximab | 17 |
|  | certolizumab pegol | 18 |
|  | abatacept | 19 |
|  | golimumab | 20 |
|  | ofatumumab | 21 |
|  | diacerein | 22 |
|  | imidazole salicylate | 23 |
|  | itolizumab | 24 |
|  | sarilumab | 25 |
|  | baricitinib | 26 |
|  | filgotinib | 27 |
|  | peficitinib | 28 |
|  | iguratimod | 29 |
|  | mizoribine | 30 |
|  | actarit | 31 |
|  | aceclofenac | 32 |
|  | hyaluronate sodium | 33 |
|  | upadacitinib | 34 |
|  | tetrachlorodecaoxide | 35 |
|  | Cartistem | 36 |
|  | once-daily naproxen | 37 |
|  | Ka Shu Ning | 38 |
|  | tirabrutinib | 39 |
|  | opinercept | 40 |
|  | Orelabrutinib | 41 |
|  | MAG-DHA | 42 |
|  | sarilumab | 43 |
|  | methotrexate | 44 |
|  | salazosulfapyridine | 45 |
|  | hydroxychloroquine | 46 |
|  | azathioprine | 47 |
|  | cyclophosphamide | 48 |
|  | sirolimus | 49 |
|  | mycophenolate mofetil | 50 |
|  | paracetamol | 51 |
|  | nimesulide | 52 |
|  | acemetacin | 53 |
|  | diclofenac potassium | 54 |
|  | esflurbiprofen | 55 |
|  | flurbiprofen | 56 |
|  | ibuprofen | 57 |
|  | indometacin | 58 |
|  | mefenamic acid | 59 |
|  | dexibuprofen | 60 |
|  | ketoprofen | 61 |
| ankylosing spondylitis | apremilast | 1 |
|  | meloxicam | 2 |
|  | celecoxib | 3 |
|  | etanercept | 4 |
|  | adalimumab | 5 |
|  | etoricoxib | 6 |
|  | infliximab | 7 |
|  | certolizumab pegol | 8 |
|  | abatacept | 9 |
|  | golimumab | 10 |
|  | secukinumab | 11 |
|  | ixekizumab | 12 |
|  | filgotinib | 13 |
|  | tildrakizumab | 14 |
|  | aceclofenac | 15 |
|  | upadacitinib | 16 |
|  | once-daily naproxen | 17 |
|  | netakimab | 18 |
|  | opinercept | 19 |
|  | SAR153191 | 20 |
|  | salazosulfapyridine | 21 |
|  | thalidomide | 22 |
|  | leflunomide | 23 |
|  | methotrexate | 24 |
|  | imrecoxib | 25 |
|  | hydroxychloroquine | 26 |
|  | paracetamol | 27 |
|  | piroxicam | 28 |
|  | nabumetone | 29 |
|  | nimesulide | 30 |
|  | acemetacin | 31 |
|  | diclofenac potassium | 32 |
|  | flurbiprofen | 33 |
|  | ibuprofen | 34 |
|  | indometacin | 35 |
|  | etodolac | 36 |
|  | dexibuprofen | 37 |
|  | ketoprofen | 38 |
| Vasculitis or systemic vasculitis | belimumab | 1 |
|  | sulfonated human immunoglobulin | 2 |
|  | methylprednisolone | 3 |
|  | cortisone | 4 |
|  | hydrocortisone | 5 |
|  | betamethasone | 6 |
|  | dexamethasone | 7 |
|  | prednisone | 8 |
|  | prednisolone | 9 |
|  | azathioprine | 10 |
|  | methotrexate | 11 |
|  | cyclophosphamide | 12 |
|  | rituximab | 13 |
|  | mycophenolate mofetil | 14 |
|  | ciclosporin | 15 |
|  | tacrolimus | 16 |
|  | atorvastatin calcium | 17 |
|  | pravastatin sodium | 18 |
|  | lovastatin | 19 |
|  | simvastatin | 20 |
|  | infliximab | 21 |
|  | triamcinolone | 22 |
| bone porosity | pamidronate disodium | 1 |
|  | tibolone | 2 |
|  | etidronic acid | 3 |
|  | raloxifene | 4 |
|  | trimegestone | 5 |
|  | zoledronic acid | 6 |
|  | minodronic acid | 7 |
|  | lasofoxifene | 8 |
|  | bazedoxifene | 9 |
|  | denosumab | 10 |
|  | eldecalcitol | 11 |
|  | romosozumab | 12 |
|  | abaloparatide | 13 |
|  | menatetrenone | 14 |
|  | strontium ranelate | 15 |
|  | risedronate sodium | 16 |
|  | alendronic acid | 17 |
|  | alfacalcidol | 18 |
|  | alendronate | 19 |
|  | medroxyprogesterone acetate | 20 |
|  | calcitriol | 21 |
|  | sodium ibandronate | 22 |
|  | Esterified Estrogen | 23 |
|  | Atelvia | 24 |
|  | calcitonin salmon | 25 |
|  | elcatonin | 26 |
|  | ethinyl estradiol | 27 |
|  | estradiol | 28 |
|  | progesterone | 29 |
|  | teriparatide | 30 |
|  | alendronate sodium | 31 |
|  | ibandronate monosodium | 32 |
|  | cyproterone acetate | 33 |
|  | colecalciferol | 34 |
| Reactive arthritis | thalidomide | 1 |
|  | sulfasalazine | 2 |
|  | aspirin | 3 |
|  | indometacin | 4 |
|  | sulindac | 5 |
|  | naproxen | 6 |
|  | ibuprofen | 7 |
|  | diclofenac sodium | 8 |
|  | paracetamol | 9 |
|  | rofecoxib | 10 |
|  | mefenamic acid | 11 |
|  | piroxicam | 12 |
|  | meloxicam | 13 |
|  | nabumetone | 14 |
|  | ofloxacin | 15 |
|  | ofloxacin | 16 |
|  | ciprofloxacin | 17 |
|  | fleroxacin | 18 |
|  | roxithromycin | 19 |
|  | azithromycin | 20 |
|  | spiramycin | 21 |
|  | tinidazole | 22 |
|  | methylprednisolone | 23 |
|  | cortisone | 24 |
|  | hydrocortisone | 25 |
|  | betamethasone | 26 |
|  | dexamethasone | 27 |
|  | prednisone | 28 |
|  | prednisolone | 29 |
|  | methotrexate | 30 |
|  | azathioprine | 31 |
|  | etanercept | 32 |
|  | adalimumab | 33 |
|  | infliximab | 34 |
|  | certolizumab pegol | 35 |
|  | golimumab | 36 |
|  | nimesulide | 37 |
|  | triamcinolone | 38 |
|  | dexibuprofen | 39 |
| Avascular Necrosis of Femoral Head | treprostinil diolamine | 1 |
|  | epoprostenol | 2 |
|  | treprostinil sodium | 3 |
|  | epoprostenol sodium | 4 |
|  | warfarin | 5 |
|  | aspirin | 6 |
|  | dextran | 7 |
|  | lumbrokinase | 8 |
|  | urokinase | 9 |
|  | atorvastatin | 10 |
|  | lovastatin | 11 |
|  | simvastatin | 12 |
|  | fluvastatin | 13 |
|  | alendronate sodium | 14 |
|  | ibandronate monosodium | 15 |
|  | calcitriol | 16 |
|  | enoxaparin sodium | 17 |
|  | edaravone | 18 |
|  | tocopherol | 19 |
|  | clopidogrel | 20 |
|  | pravastatin sodium | 21 |
| scleroderma | belimumab | 1 |
|  | pomalidomide | 2 |
|  | bosentan | 3 |
|  | riociguat | 4 |
|  | hyaluronidase | 5 |
|  | dimethyl fumarate | 6 |
|  | mycophenolate mofetil | 7 |
|  | elapegademase | 8 |
|  | Privigen | 9 |
|  | Hizentra | 10 |
|  | tagraxofusp | 11 |
|  | methylprednisolone | 12 |
|  | cortisone | 13 |
|  | hydrocortisone | 14 |
|  | betamethasone | 15 |
|  | dexamethasone | 16 |
|  | prednisone | 17 |
|  | prednisolone | 18 |
|  | triamcinolone acetonide | 19 |
|  | triamcinolone | 20 |
|  | ciclosporin | 21 |
|  | methotrexate | 22 |
|  | cyclophosphamide | 23 |
|  | mycophenolate mofetil | 24 |
|  | azathioprine | 25 |
|  | penicillamine | 26 |
|  | colchicine | 27 |
|  | sodium calcium edetate | 28 |
|  | nisoldipine | 29 |
|  | benidipine | 30 |
|  | lacidipine | 31 |
|  | lidoflazine | 32 |
|  | flunarizine | 33 |
|  | verapamil | 34 |
|  | diltiazem | 35 |
|  | nimodipine | 36 |
|  | lercanidipine | 37 |
|  | amlodipine | 38 |
|  | nifedipine | 39 |
|  | prazosin | 40 |
|  | treprostinil diolamine | 41 |
|  | epoprostenol | 42 |
|  | treprostinil sodium | 43 |
|  | epoprostenol sodium | 44 |
|  | losartan | 45 |
|  | quinapril | 46 |
|  | bosentan | 47 |
|  | macitentan | 48 |
|  | ambrisentan | 49 |
|  | sildenafil | 50 |
|  | tadalafil | 51 |
|  | fluoxetine | 52 |
|  | interferon gamma | 53 |
|  | rituximab | 54 |
|  | infliximab | 55 |
|  | quinidine | 56 |
|  | propranolol | 57 |
|  | verapamil | 58 |
|  | amiodarone | 59 |
|  | adenosine triphosphate | 60 |
|  | cobamamide | 61 |
|  | tacrolimus | 62 |
|  | tocilizumab | 63 |
|  | dabigatran etexilate | 64 |
| Psoriatic arthritis | apremilast | 1 |
|  | leflunomide | 2 |
|  | etanercept | 3 |
|  | adalimumab | 4 |
|  | infliximab | 5 |
|  | certolizumab pegol | 6 |
|  | golimumab | 7 |
|  | ustekinumab | 8 |
|  | secukinumab | 9 |
|  | itolizumab | 10 |
|  | ixekizumab | 11 |
|  | brodalumab | 12 |
|  | baricitinib | 13 |
|  | filgotinib | 14 |
|  | tildrakizumab | 15 |
|  | guselkumab | 16 |
|  | risankizumab | 17 |
|  | upadacitinib | 18 |
|  | netakimab | 19 |
|  | methotrexate | 20 |
|  | salazosulfapyridine | 21 |
|  | tofacitinib | 22 |
|  | tofacitinib citrate | 23 |
|  | diclofenac potassium | 24 |
|  | diclofenac sodium | 25 |
|  | diclofenac | 26 |
|  | meloxicam | 27 |
|  | ibuprofen | 28 |
|  | dexibuprofen | 29 |
|  | flurbiprofen | 30 |
|  | celecoxib | 31 |
|  | ciclosporin | 32 |
|  | methylprednisolone | 33 |
|  | cortisone | 34 |
|  | hydrocortisone | 35 |
|  | betamethasone | 36 |
|  | dexamethasone | 37 |
|  | prednisone | 38 |
|  | prednisolone | 39 |
|  | triamcinolone acetonide | 40 |
|  | triamcinolone | 41 |
|  | ustekinumab | 42 |
|  | abatacept | 43 |
|  | secukinumab | 44 |
|  | ixekizumab | 45 |
|  | brodalumab | 46 |
| Fibromyalgia | memantine hydrochloride | 1 |
|  | milnacipran | 2 |
|  | mirtazapine | 3 |
|  | duloxetine | 4 |
|  | pregabalin | 5 |
|  | fremanezumab | 6 |
|  | amitriptyline | 7 |
|  | tramadol | 8 |
|  | cyclobenzaprine | 9 |
|  | fluoxetine | 10 |
|  | paroxetine | 11 |
|  | citalopram | 12 |
|  | sertraline hydrochloride | 13 |
|  | milnacipran | 14 |
|  | venlafaxine | 15 |
|  | moclobemide | 16 |
|  | pramipexole | 17 |
|  | zolpidem | 18 |
|  | tropisetron | 19 |
|  | trazodone hydrochloride | 20 |
|  | celecoxib | 21 |
|  | aspirin | 22 |
|  | indometacin | 23 |
|  | sulindac | 24 |
|  | naproxen | 25 |
|  | ibuprofen | 26 |
|  | diclofenac sodium | 27 |
|  | paracetamol | 28 |
|  | rofecoxib | 29 |
|  | mefenamic acid | 30 |
|  | piroxicam | 31 |
|  | meloxicam | 32 |
|  | nabumetone | 33 |
|  | famciclovir | 34 |
|  | thioctic acid | 35 |
|  | dexibuprofen | 36 |
|  | ketoprofen | 37 |
| Systemic lupus erythematosus | belimumab | 1 |
|  | pomalidomide | 2 |
|  | bosentan | 3 |
|  | riociguat | 4 |
|  | ustekinumab | 5 |
|  | baricitinib | 6 |
|  | bovhyaluronidase azoximer | 7 |
|  | dimethyl fumarate | 8 |
|  | mycophenolate mofetil | 9 |
|  | elapegademase | 10 |
|  | upadacitinib | 11 |
|  | Privigen | 12 |
|  | Hizentra | 13 |
|  | tirabrutinib | 14 |
|  | sirolimus | 15 |
|  | hydroxychloroquine | 16 |
|  | chloroquine | 17 |
|  | aspirin | 18 |
|  | indometacin | 19 |
|  | sulindac | 20 |
|  | naproxen | 21 |
|  | ibuprofen | 22 |
|  | diclofenac sodium | 23 |
|  | paracetamol | 24 |
|  | rofecoxib | 25 |
|  | mefenamic acid | 26 |
|  | piroxicam | 27 |
|  | meloxicam | 28 |
|  | nabumetone | 29 |
|  | nimesulide | 30 |
|  | methylprednisolone | 31 |
|  | cortisone | 32 |
|  | hydrocortisone | 33 |
|  | betamethasone | 34 |
|  | dexamethasone | 35 |
|  | prednisone | 36 |
|  | prednisolone | 37 |
|  | triamcinolone acetonide | 38 |
|  | triamcinolone | 39 |
|  | mycophenolate mofetil | 40 |
|  | cyclophosphamide | 41 |
|  | leflunomide | 42 |
|  | methotrexate | 43 |
|  | tacrolimus | 44 |
|  | ciclosporin | 45 |
|  | azathioprine | 46 |
|  | belimumab | 47 |
|  | rituximab | 48 |
|  | mizoribine | 49 |
|  | celecoxib | 50 |
|  | dexibuprofen | 51 |
|  | ketoprofen | 52 |
| Diabetic peripheral neuropathy | duloxetine | 1 |
|  | thioctic acid | 2 |
|  | reduced glutathione | 3 |
|  | pentoxifylline | 4 |
|  | nisoldipine | 5 |
|  | benidipine | 6 |
|  | lacidipine | 7 |
|  | lidoflazine | 8 |
|  | flunarizine | 9 |
|  | verapamil | 10 |
|  | diltiazem | 11 |
|  | nimodipine | 12 |
|  | lercanidipine | 13 |
|  | amlodipine | 14 |
|  | nifedipine | 15 |
|  | mecobalamin | 16 |
|  | inositol | 17 |
|  | gamolenic acid | 18 |
|  | venlafaxine | 19 |
|  | pregabalin | 20 |
|  | carbamazepine | 21 |
|  | gabapentin | 22 |
|  | sodium valproate | 23 |
|  | tramadol | 24 |
|  | oxycodone hydrochloride | 25 |
|  | capsaicin | 26 |
|  | cyanocobalamin | 27 |
| Dysmenorrhea | celecoxib | 1 |
|  | lumiracoxib | 2 |
|  | once-daily naproxen | 3 |
|  | dienogest | 4 |
|  | ibuprofen | 5 |
|  | ketoprofen | 6 |
|  | diclofenac sodium | 7 |
|  | mefenamic acid | 8 |
|  | naproxen | 9 |
|  | cyproterone acetate | 10 |
|  | desogestrel | 11 |
|  | ethinyl estradiol | 12 |
|  | medroxyprogesterone acetate | 13 |
|  | pyridoxinium chloride | 14 |
|  | clonidine | 15 |
|  | menadione sodium bisulfite | 16 |
|  | atropine | 17 |
|  | diazepam | 18 |
|  | nifedipine | 19 |
|  | isoprenaline | 20 |
|  | meloxicam | 21 |
|  | paracetamol | 22 |
|  | piroxicam | 23 |
|  | nabumetone | 24 |
|  | rofecoxib | 25 |
|  | nimesulide | 26 |
|  | etoricoxib | 27 |
|  | aceclofenac | 28 |
|  | acemetacin | 29 |
|  | diclofenac potassium | 30 |
|  | flurbiprofen | 31 |
|  | parecoxib | 32 |
|  | indometacin | 33 |
|  | etodolac | 34 |
|  | dexibuprofen | 35 |
|  | estradiol | 36 |
| spinal stenosis | limaprost | 1 |
|  | selexipag | 2 |
|  | calcitonin salmon | 3 |
|  | elcatonin | 4 |
|  | mecobalamin | 5 |
|  | alprostadil | 6 |
|  | gabapentin | 7 |
|  | cyanocobalamin | 8 |
| Sjogren's syndrome | belimumab | 1 |
|  | filgotinib | 2 |
|  | tirabrutinib | 3 |
|  | paracetamol | 4 |
|  | gabapentin | 5 |
|  | pregabalin | 6 |
|  | duloxetine | 7 |
|  | methylprednisolone | 8 |
|  | cortisone | 9 |
|  | hydrocortisone | 10 |
|  | betamethasone | 11 |
|  | dexamethasone | 12 |
|  | prednisone | 13 |
|  | prednisolone | 14 |
|  | triamcinolone acetonide | 15 |
|  | triamcinolone | 16 |
|  | hydroxychloroquine | 17 |
|  | methotrexate | 18 |
|  | leflunomide | 19 |
|  | mycophenolate mofetil | 20 |
|  | azathioprine | 21 |
|  | cyclophosphamide | 22 |
|  | ciclosporin | 23 |
|  | iguratimod | 24 |
|  | tacrolimus | 25 |
|  | rituximab | 26 |
|  | thiamine | 27 |
|  | cyanocobalamin | 28 |
|  | mizoribine | 29 |
|  | mecobalamin | 30 |

**Supplementary Table 7.** Western medicines with high cluster similarity and their classification.

| Drug | GO group | WP group | REAC group | ATC classification |
| --- | --- | --- | --- | --- |
| etidronic acid | 4 | 3 | 5 | Affecting bone mineralization |
| alendronate sodium | 4 | 3 | 5 | Affecting bone mineralization |
| risedronate sodium | 4 | 3 | 5 | Affecting bone mineralization |
| pamidronate disodium | 4 | 3 | 5 | Affecting bone structure |
| zoledronic acid | 4 | 3 | 5 | Affecting bone structure |
| minodronic acid | 4 | 3 | 5 | Affecting bone structure |
| ibandronate monosodium | 4 | 3 | 5 | Affecting bone structure |
| eldecalcitol | 5 | 1 | 5 | Affecting bone structure |
| menatetrenone | 8 | 2 | 4 | Affecting bone structure |
| adenosine triphosphate | 1 | 2 | 4 | antiarrhythmics |
| quinidine | 8 | 6 | 4 | antiarrhythmics |
| isoprenaline | 8 | 2 | 4 | antiarrhythmics |
| atropine | 11 | 6 | 10 | antiarrhythmics |
| procainamide | 14 | 18 | 13 | antiarrhythmics |
| propranolol | 8 | 6 | 6 | antiarrhythmics |
| amiodarone | 8 | 6 | 4 | antiarrhythmics |
| azithromycin | 9 | 1 | 5 | Antibacterials-1 |
| roxithromycin | 9 | 1 | 5 | Antibacterials-1 |
| spiramycin | 9 | 1 | 5 | Antibacterials-1 |
| fleroxacin | 16 | 17 | 9 | Antibacterials-2 |
| ciprofloxacin | 16 | 17 | 15 | Antibacterials-2 |
| ofloxacin | 16 | 17 | 9 | Antibacterials-2 |
| febuxostat | 6 | 4 | 11 | antigout preparations |
| allopurinol | 6 | 4 | 5 | antigout preparations |
| benzbromarone | 9 | 13 | 2 | antigout preparations |
| topiroxostat | 13 | 12 | 12 | antigout preparations |
| sodium bicarbonate | 15 | 16 | 13 | antigout preparations |
| colchicine | 18 | 7 | 16 | antigout preparations |
| bosentan | 7 | 5 | 7 | antihypertensives |
| macitentan | 7 | 5 | 7 | antihypertensives |
| ambrisentan | 7 | 5 | 7 | antihypertensives |
| prazosin | 8 | 6 | 6 | antihypertensives |
| nitroprusside sodium | 7 | 20 | 18 | antihypertensives |
| clonidine | 8 | 6 | 6 | antihypertensives |
| penicillamine | 1 | 2 | 1 | antirheumatic |
| actarit | 3 | 1 | 3 | antirheumatic |
| salazosulfapyridine | 3 | 1 | 1 | antirheumatic |
| hydroxychloroquine | 3 | 1 | 12 | antirheumatic |
| glucosamine | 3 | 1 | 3 | antirheumatic |
| leflunomide | 3 | 10 | 5 | antirheumatic |
| chloroquine | 8 | 14 | 10 | antirheumatic |
| thalidomide | 8 | 2 | 4 | antirheumatic |
| azathioprine | 12 | 11 | 11 | antirheumatic |
| diacerein | 19 | 19 | 17 | antirheumatic |
| warfarin | 2 | 10 | 2 | Antithrombotic agents |
| treprostinil diolamine | 5 | 1 | 3 | Antithrombotic agents |
| cilostazol | 7 | 9 | 7 | Antithrombotic agents |
| argatroban | 7 | 8 | 7 | Antithrombotic agents |
| dabigatran etexilate | 7 | 8 | 7 | Antithrombotic agents |
| dextran | 9 | 1 | 5 | Antithrombotic agents |
| apixaban | 10 | 8 | 9 | Antithrombotic agents |
| rivaroxaban | 10 | 8 | 9 | Antithrombotic agents |
| epoprostenol | 15 | 5 | 7 | Antithrombotic agents |
| clopidogrel | 15 | 5 | 7 | Antithrombotic agents |
| sodium citrate | 16 | 17 | 9 | Antithrombotic agents |
| reduced glutathione | 1 | 2 | 1 | Antitumor drugs |
| masitinib | 7 | 9 | 6 | Antitumor drugs |
| nifedipine | 8 | 6 | 4 | calcium channel blockers |
| nilvadipine | 8 | 6 | 4 | calcium channel blockers |
| nisoldipine | 8 | 6 | 4 | calcium channel blockers |
| amlodipine | 8 | 6 | 4 | calcium channel blockers |
| nimodipine | 8 | 6 | 4 | calcium channel blockers |
| lacidipine | 8 | 6 | 4 | calcium channel blockers |
| verapamil | 8 | 6 | 4 | calcium channel blockers |
| benidipine | 8 | 6 | 4 | calcium channel blockers |
| lercanidipine | 8 | 6 | 4 | calcium channel blockers |
| diltiazem | 15 | 16 | 13 | calcium channel blockers |
| calcitonin salmon | 7 | 6 | 7 | calcium homeostasis |
| elcatonin | 7 | 6 | 7 | calcium homeostasis |
| teriparatide | 7 | 6 | 7 | calcium homeostasis |
| cobamamide | 1 | 18 | 1 | Cardiovascular System |
| prednisone | 3 | 1 | 3 | corticosteroid |
| triamcinolone | 3 | 1 | 3 | corticosteroid |
| prednisolone | 3 | 1 | 3 | corticosteroid |
| cortisone | 3 | 1 | 3 | corticosteroid |
| hydrocortisone | 3 | 1 | 3 | corticosteroid |
| betamethasone | 3 | 1 | 3 | corticosteroid |
| dexamethasone | 3 | 1 | 3 | corticosteroid |
| methylprednisolone | 3 | 1 | 3 | corticosteroid |
| deflazacort | 5 | 1 | 15 | corticosteroid |
| cortisone acetate | 5 | 1 | 3 | corticosteroid |
| interferon gamma | 16 | 17 | 9 | Immunomodulatory |
| methotrexate | 2 | 1 | 2 | Immunosuppressant-1 |
| tacrolimus | 3 | 12 | 3 | Immunosuppressant-1 |
| sirolimus | 3 | 12 | 3 | Immunosuppressant-1 |
| ciclosporin | 5 | 16 | 12 | Immunosuppressant-1 |
| apremilast | 7 | 9 | 6 | Immunosuppressant-1 |
| pomalidomide | 8 | 2 | 4 | Immunosuppressant-1 |
| anakinra | 9 | 7 | 8 | Immunosuppressant-1 |
| mycophenolate mofetil | 12 | 11 | 11 | Immunosuppressant-2 |
| mizoribine | 12 | 11 | 11 | Immunosuppressant-2 |
| tofacitinib | 13 | 12 | 12 | Immunosuppressant-2 |
| baricitinib | 13 | 12 | 12 | Immunosuppressant-2 |
| eperisone | 8 | 12 | 6 | Muscle relaxants |
| phenprobamate | 11 | 9 | 10 | Muscle relaxants |
| chlorzoxazone | 15 | 16 | 13 | Muscle relaxants |
| cyclobenzaprine | 17 | 6 | 6 | Muscle relaxants |
| hyaluronate sodium | 10 | 8 | 9 | musculoskeletal system |
| trazodone hydrochloride | 17 | 6 | 6 | nervous system |
| moclobemide | 14 | 18 | 7 | nervous system |
| diazepam | 8 | 14 | 10 | nervous system |
| capsaicin | 15 | 15 | 14 | nervous system |
| memantine hydrochloride | 11 | 9 | 10 | nervous system |
| vinpocetine | 7 | 9 | 6 | nervous system |
| edaravone | 8 | 2 | 4 | nervous system |
| gabapentin | 1 | 2 | 1 | nervous system-1 |
| sodium valproate | 1 | 2 | 1 | nervous system-1 |
| ademetionine | 1 | 2 | 4 | nervous system-1 |
| mirtazapine | 8 | 6 | 4 | nervous system-2 |
| venlafaxine | 8 | 6 | 4 | nervous system-2 |
| milnacipran | 11 | 9 | 10 | nervous system-2 |
| amitriptyline | 8 | 6 | 4 | nervous system-2 |
| pramipexole | 8 | 6 | 6 | nervous system-3 |
| flunarizine | 8 | 6 | 6 | nervous system-3 |
| fosphenytoin sodium | 8 | 2 | 4 | nervous system-3 |
| carbamazepine | 14 | 14 | 13 | nervous system-3 |
| lidocaine | 8 | 6 | 4 | nervous system-3 |
| ketamine | 8 | 6 | 4 | nervous system-3 |
| bupivacaine | 14 | 14 | 13 | nervous system-3 |
| zolpidem | 8 | 2 | 4 | nervous system-3 |
| pregabalin | 1 | 2 | 1 | nervous system-4 |
| tramadol | 8 | 2 | 4 | nervous system-4 |
| pethidine | 11 | 6 | 10 | nervous system-4 |
| hydromorphone hydrochloride | 11 | 6 | 6 | nervous system-4 |
| oxycodone hydrochloride | 11 | 6 | 6 | nervous system-4 |
| fentanyl | 11 | 6 | 6 | nervous system-4 |
| buprenorphine | 11 | 6 | 6 | nervous system-4 |
| duloxetine | 17 | 10 | 8 | nervous system-5 |
| tropisetron | 8 | 10 | 8 | nervous system-5 |
| fluoxetine | 11 | 9 | 10 | nervous system-5 |
| sertraline hydrochloride | 17 | 10 | 8 | nervous system-5 |
| citalopram | 17 | 10 | 8 | nervous system-5 |
| paroxetine | 17 | 6 | 6 | nervous system-5 |
| aspirin | 1 | 2 | 1 | non-steroids-1 |
| naproxen | 1 | 1 | 1 | non-steroids-1 |
| tenoxicam | 2 | 1 | 2 | non-steroids-1 |
| nimesulide | 2 | 1 | 2 | non-steroids-1 |
| diclofenac potassium | 2 | 1 | 2 | non-steroids-1 |
| meloxicam | 2 | 1 | 2 | non-steroids-1 |
| flurbiprofen | 2 | 1 | 2 | non-steroids-1 |
| piroxicam | 2 | 1 | 2 | non-steroids-1 |
| nabumetone | 2 | 1 | 2 | non-steroids-1 |
| aceclofenac | 2 | 1 | 2 | non-steroids-1 |
| celecoxib | 2 | 1 | 2 | non-steroids-1 |
| rofecoxib | 2 | 1 | 2 | non-steroids-1 |
| etoricoxib | 2 | 1 | 2 | non-steroids-1 |
| parecoxib | 2 | 1 | 2 | non-steroids-1 |
| sulindac | 2 | 1 | 6 | non-steroids-1 |
| lumiracoxib | 2 | 1 | 2 | non-steroids-1 |
| acemetacin | 2 | 1 | 2 | non-steroids-1 |
| paracetamol | 2 | 1 | 2 | non-steroids-1 |
| esflurbiprofen | 2 | 1 | 2 | non-steroids-1 |
| pelubiprofen | 2 | 1 | 2 | non-steroids-1 |
| dexibuprofen | 3 | 1 | 3 | non-steroids-2 |
| mefenamic acid | 3 | 1 | 3 | non-steroids-2 |
| ketoprofen | 3 | 1 | 3 | non-steroids-2 |
| etodolac | 3 | 1 | 2 | non-steroids-2 |
| indometacin | 3 | 1 | 3 | non-steroids-2 |
| ibuprofen | 3 | 1 | 3 | non-steroids-2 |
| imidazole salicylate | 3 | 1 | 1 | non-steroids-2 |
| benzydamine hydrochloride | 8 | 2 | 4 | non-steroids-2 |
| citicoline | 1 | 2 | 4 | Nutrition |
| aniracetam | 2 | 1 | 2 | Nutrition |
| oxiracetam | 15 | 16 | 19 | Nutrition |
| gamolenic acid | 1 | 1 | 1 | Nutrition |
| mecobalamin | 1 | 18 | 1 | Nutrition |
| trihexyphenidyl | 17 | 6 | 6 | Nutrition |
| ergocalciferol | 1 | 1 | 1 | Nutrition |
| cyanocobalamin | 1 | 18 | 1 | Nutrition |
| MAG-DHA | 1 | 1 | 1 | Other types |
| limaprost | 3 | 1 | 6 | Peripheral vasodilator |
| pentoxifylline | 8 | 2 | 4 | Peripheral vasodilator |
| telmisartan | 3 | 1 | 3 | Renin-angiotensin system |
| losartan | 13 | 19 | 10 | Renin-angiotensin system |
| quinapril | 19 | 19 | 17 | Renin-angiotensin system |
| trimegestone | 1 | 1 | 1 | sex hormones-1 |
| dienogest | 5 | 1 | 3 | sex hormones-1 |
| tibolone | 5 | 1 | 3 | sex hormones-1 |
| desogestrel | 5 | 1 | 3 | sex hormones-1 |
| cyproterone acetate | 5 | 1 | 3 | sex hormones-1 |
| medroxyprogesterone acetate | 5 | 1 | 3 | sex hormones-1 |
| lasofoxifene | 5 | 1 | 15 | sex hormones-1 |
| progesterone | 8 | 2 | 4 | sex hormones-1 |
| estradiol | 11 | 1 | 3 | sex hormones-2 |
| ethinyl estradiol | 11 | 1 | 3 | sex hormones-2 |
| raloxifene | 11 | 1 | 3 | sex hormones-2 |
| furosemide | 8 | 14 | 10 | urologicals |
| mannitol | 11 | 5 | 14 | urologicals |
| potassium citrate | 16 | 17 | 9 | urologicals |
| tadalafil | 20 | 9 | 20 | urologicals |
| sildenafil | 20 | 9 | 20 | urologicals |
| alprostadil | 3 | 1 | 6 | Vasodilators |
| sodium aescinate | 3 | 1 | 15 | Vasodilators |
| nicotinic acid | 3 | 1 | 3 | Vasodilators |
| atorvastatin | 2 | 10 | 5 | Vasodilators |
| fluvastatin | 2 | 10 | 5 | Vasodilators |
| rosuvastatin | 2 | 3 | 5 | Vasodilators |
| pravastatin sodium | 3 | 3 | 15 | Vasodilators |
| lovastatin | 3 | 3 | 15 | Vasodilators |
| simvastatin | 3 | 3 | 15 | Vasodilators |
| bezafibrate | 3 | 1 | 3 | Vasodilators |
| ciprofibrate | 5 | 1 | 5 | Vasodilators |
| fenofibrate | 5 | 1 | 15 | Vasodilators |
| ezetimibe | 19 | 3 | 17 | Vasodilators |
| tocopherol | 3 | 12 | 6 | vitamins |
| colecalciferol | 5 | 1 | 5 | vitamins |
| alfacalcidol | 5 | 1 | 5 | vitamins |
| calcitriol | 5 | 1 | 5 | vitamins |
| thioctic acid | 9 | 10 | 2 | vitamins |
| thiamine | 9 | 10 | 2 | vitamins |
| inositol | 11 | 5 | 14 | vitamins |
| pyridoxinium chloride | 12 | 17 | 2 | vitamins |

**Supplementary Table 8.** Ranking data of YXB's indications based on similarity analysis results.

| Disease name | MatchedDrugGroup | | | DrugNum | | | MatchedDrugType | | |
| --- | --- | --- | --- | --- | --- | --- | --- | --- | --- |
|  | Matched | Unmatched | MatchedGroupRatio | Matched | Unmatched | MatchedNumRatio | Matched | Unmatched | MatchedTypeRatio |
| Psoriatic arthritis | 4 | 0 | 1.00 | 13 | 2 | 0.87 | 4 | 0 | 1.00 |
| Dysmenorrhea | 8 | 0 | 1.00 | 21 | 6 | 0.78 | 5 | 0 | 1.00 |
| Cervical spondylosis | 8 | 0 | 1.00 | 26 | 7 | 0.79 | 6 | 1 | 0.86 |
| Lumbar disc herniation | 7 | 0 | 1.00 | 23 | 9 | 0.72 | 6 | 1 | 0.86 |
| Swelling and pain after fracture | 9 | 0 | 1.00 | 20 | 10 | 0.67 | 6 | 2 | 0.75 |
| Stroke prevention | 5 | 0 | 1.00 | 8 | 6 | 0.57 | 4 | 1 | 0.80 |
| Osteoarthritis | 7 | 1 | 0.88 | 21 | 14 | 0.60 | 5 | 2 | 0.71 |
| Frozen shoulder | 6 | 1 | 0.86 | 22 | 5 | 0.81 | 3 | 0 | 1.00 |
| Tenosynovitis | 6 | 1 | 0.86 | 21 | 5 | 0.81 | 3 | 0 | 1.00 |
| Osteoporosis | 5 | 1 | 0.83 | 15 | 5 | 0.75 | 5 | 1 | 0.83 |
| Ankylosing spondylitis | 4 | 1 | 0.80 | 12 | 6 | 0.67 | 3 | 0 | 1.00 |
| Fibromyalgia | 4 | 1 | 0.80 | 9 | 10 | 0.47 | 4 | 1 | 0.80 |
| Reactive arthritis | 7 | 2 | 0.78 | 20 | 5 | 0.80 | 5 | 0 | 1.00 |
| Vasculitis or systemic vasculitis | 3 | 1 | 0.75 | 10 | 3 | 0.77 | 3 | 1 | 0.75 |
| gout | 6 | 2 | 0.75 | 22 | 7 | 0.76 | 3 | 1 | 0.75 |
| Rheumatoid arthritis | 5 | 2 | 0.71 | 20 | 10 | 0.67 | 4 | 1 | 0.80 |
| Sjogren's syndrome | 5 | 2 | 0.71 | 11 | 6 | 0.65 | 4 | 4 | 0.50 |
| scleroderma | 5 | 2 | 0.71 | 16 | 11 | 0.59 | 4 | 4 | 0.50 |
| Diabetic peripheral neuropathy | 4 | 2 | 0.67 | 11 | 6 | 0.65 | 6 | 1 | 0.86 |
| Avascular Necrosis of Femoral Head | 2 | 1 | 0.67 | 3 | 3 | 0.50 | 3 | 1 | 0.75 |
| Systemic lupus erythematosus | 5 | 3 | 0.63 | 16 | 10 | 0.62 | 4 | 3 | 0.57 |
| Spinal stenosis | 3 | 2 | 0.60 | 5 | 2 | 0.71 | 3 | 1 | 0.75 |
| Stroke and sequelae of cerebral infarction | 1 | 1 | 0.50 | 1 | 1 | 0.50 | 1 | 1 | 0.50 |
| Stroke | 1 | 1 | 0.50 | 1 | 3 | 0.25 | 1 | 1 | 0.50 |
